# Controlling chemoselectivity of heterogeneous Pt catalysts *via* a support induced effect

**DOI:** 10.1039/d6cy00493h

**Published:** 2026-09-29

**Authors:** Lifeng Xiao, Kushagra Agrawal, Rongsheng Cai, Ruiyang Qu, Sofia Mediavilla Madrigal, Matthew Conway, David J. Morgan, Stuart H. Taylor, Andrew M. Beale, Sarah J. Haigh, Andrew J. Logsdail, Meenakshisundaram Sankar

**Affiliations:** a Cardiff Catalysis Institute, School of Chemistry, Translational Research Hub, Cardiff University Maindy Road Cardiff CF24 4HQ UK sankar@cardiff.ac.uk; b Department of Materials, University of Manchester Manchester M13 9PL UK; c Leibniz-Institut für Katalyse e.V. Albert-Einstein-Straße 29a 18059 Rostock Germany; d CHN Energy Europe Research GmbH Englische Str. 21 Berlin 10587 Germany; e Chemistry Department, University College London 20 Gordon Street London WC1H 0AJ UK; f Research Complex at Harwell, Rutherford Appleton Laboratory Didcot OX110FA UK

## Abstract

Tuning the chemoselectivity of a supported metal heterogeneous catalyst during hydrogenation reactions is extremely important and challenging, often achieved by changing the metal or adding homogeneous additives to the reaction. Tuning of the chemoselectivity for a given reaction over a supported metal catalyst, with a better understanding of the nature of the active site, is highly valuable to both academia and industry. Here, we report a support induced chemoselectivity (SIC) switch between Pt/TiO_2_ and Pt/Nb_2_O_5_ during the hydrogenation of 3-nitrostyrene with high chemoselectivity. Specifically, 0.2%Pt/TiO_2_ selectively reduces the –NO_2_ group to form 3-vinyl aniline, whereas 0.2%Pt/Nb_2_O_5_ selectively reduces the C=C bond to form 3-ethylnitrobenzene. Advanced characterisation and *ab initio* calculations indicate that the observed differences in selectivity could be because of support-dependent interactions at Pt–support interfacial sites, which modify the local electronic environment and adsorption behaviour of the substrate. These results suggest that the support–metal interfacial sites could potentially play a key role in controlling and switching chemoselectivity during 3-nitrostyrene hydrogenation. These results demonstrate that, in addition to modifying the metallic sites, the chemoselectivity of supported Pt nanoparticles can also be tuned by changing the support, offering an additional approach for designing new chemoselective supported metal catalysts for 3-nitrostyrene hydrogenation.

## Abstract 2

Our combined experimental and computational investigations demonstrate that besides metallic sites, metal–support interfacial sites could also play a key role in the chemoselective hydrogenation of 3-nitrostyrene.

## Graphical abstract

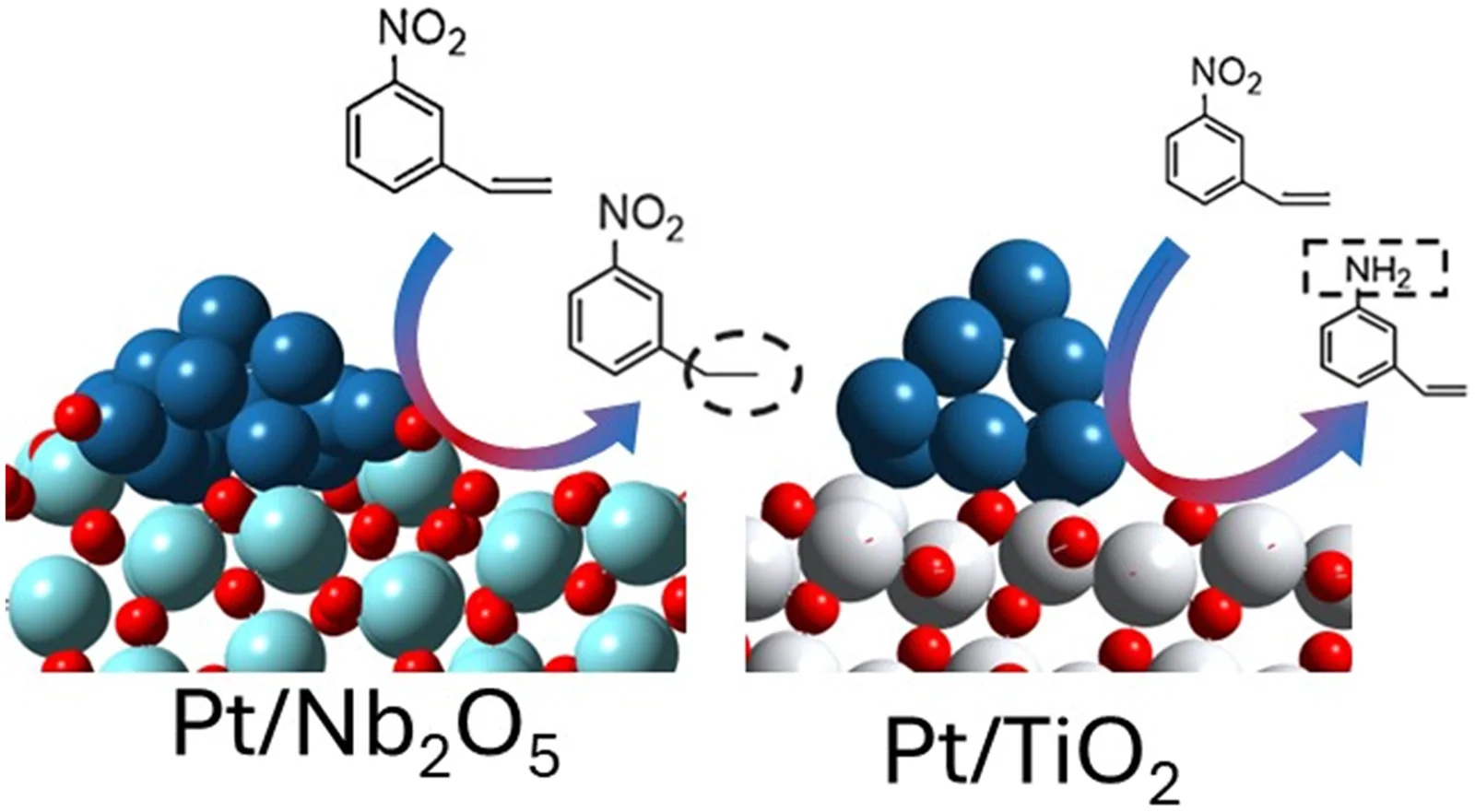

## Introduction

Selective hydrogenation reactions using supported metal nanoparticles are an important class of reactions in both industry and academia,^1^ with ∼25% of all industrial chemical transformations estimated to involve a hydrogenation step.^2^ An important example is the conversion of nitroarenes to anilines, which is crucial in the production of fine chemicals, pharmaceuticals, biologically active compounds, and numerous bulk chemicals such as polymers, fertilizers, and dyes.^1,3–5^ Traditionally, the industrial-scale reduction of nitroarenes uses stoichiometric reducing agents such as NaHSO_3_, Fe and HCl (the Bechamp process), SnCl_2_, SmI_2_, or Zn and NH_4_OH, which results in the generation of significant quantities of waste.^6,7^ A more sustainable alternative is the catalytic hydrogenation of nitroarenes using molecular H_2_, where the only by-product is water, making the chemical process environmentally benign.^1^ The challenge for this reduction reaction is to achieve chemoselectivity, *i.e.*, reducing only the target functional group and preserving all other reducible groups. For instance, chemoselectively reducing nitroarenes to functionalized anilines in the presence of reducible functional groups, such as C=O, C=C, C≡C, and C≡N, is crucial for industrial applications due to the high commercial value of the resultant products.^3–5^ Designing a chemoselective heterogeneous catalyst requires the identification of active sites on the catalyst surface, a task that is extremely challenging.

Typically, 3-nitrostyrene (3-NS) is used as a model substrate for developing heterogeneous catalysts for chemo selective hydrogenation because it contains two reducible groups: nitro and vinyl.^5,8–11^ Two competing reduction reactions are possible: hydrogenation of 3-NS to 3-vinylaniline (3-VA), where the –NO_2_ group is hydrogenated to an –NH_2_ group, and hydrogenation of 3-NS to 3-ethylnitrobenzene (3-ENB), where the C=C group is hydrogenated to ethyl group. The products of these two reactions, 3-VA and 3-ENB, can be further hydrogenated to form 3-ethylaniline (3-EA) (Scheme 1). The apparent activation energies for the hydrogenation of nitrobenzene (26–67 kJ mol^−1^) and styrene (23–77 kJ mol^−1^) are similar, making it extremely challenging to tune the chemoselectivity between these two hydrogenation reactions.^12^

**Scheme 1:**
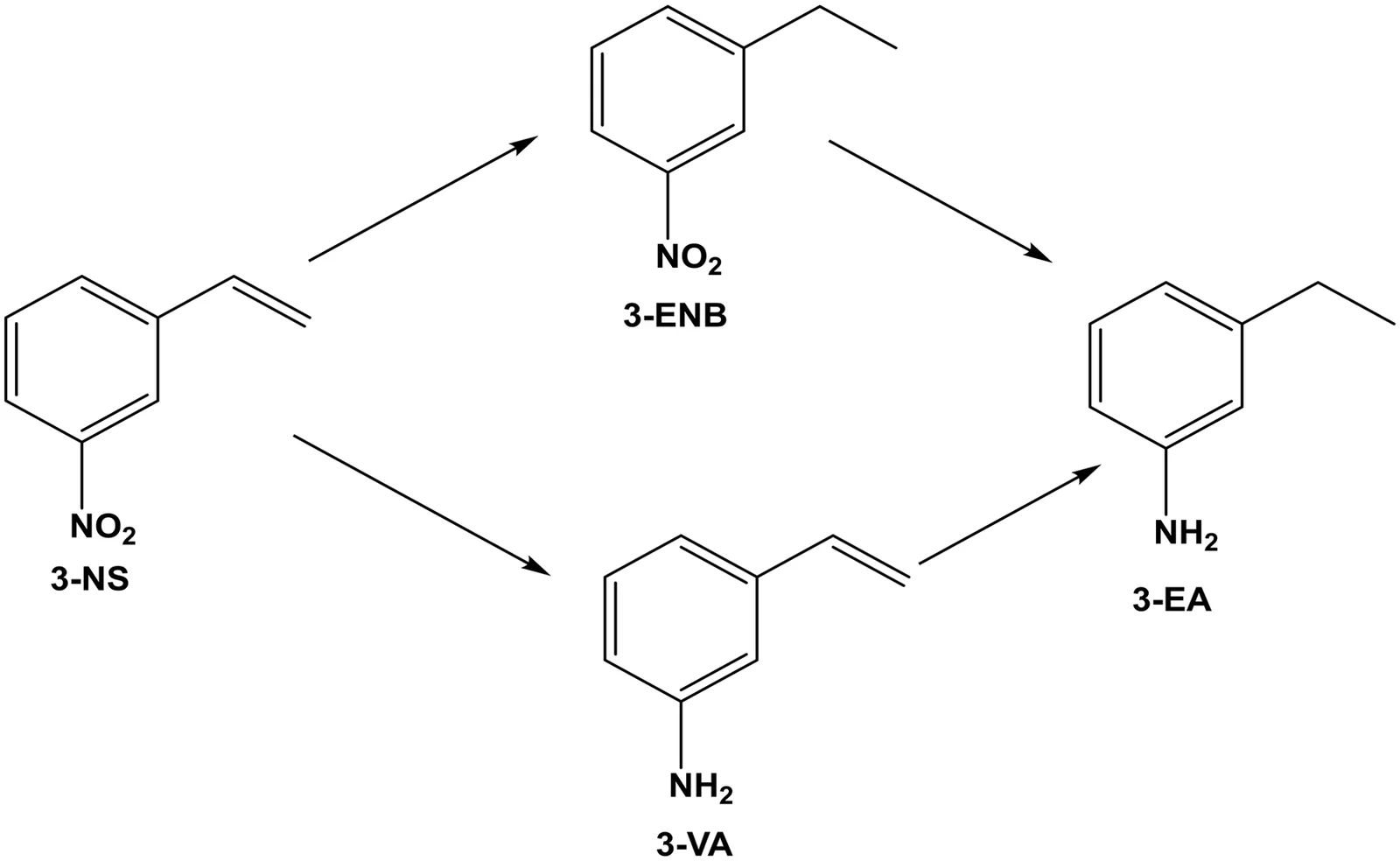
Hydrogenation pathways of 3-nitrostyrene (3-NS) to 3-ethylaniline (3-EA). 3-VA: 3-vinlyaniline; 3-ENB: 1-ethyl-3-nitrobenzene; 3-EA: 3-ethylaniline.

On the assumption that the active sites for this hydrogenation reaction are nanoparticle metallic sites, several strategies have been reported to tune the chemoselectivity of supported metal catalysts for the hydrogenation of 3-NS. Changing the metal is an obvious choice; for example, supported gold^3,13,14^ and silver^15,16^ catalysts selectively reduce the nitro group in the presence of other reducible groups (*e.g.*, CN, C=C and CHO) to produce functionalized amines. However, these metals are intrinsically less capable of activating hydrogen,^17^ making them less active for the reaction. Typical hydrogenation catalysts, such as supported Pt, Pd, and Ru nanoparticles, exhibit high catalytic activity, but the selectivity towards 3-VA is lower.^1^ Supported Pd nanoparticles, in particular, are well known for selectively hydrogenating C=C and C≡C bonds.^18^

Corma *et al.* investigated the role of support in tuning chemoselectivity during the hydrogenation of 3-NS to 3-VA, and they demonstrated that enhancing the strong metal–support interaction (SMSI) can transform a non-selective Pt/TiO_2_ catalyst into a selective one. The unselective Pt/TiO_2_ catalyst produced a mixture of 3-VA and 3-EA, with 3-EA being the fully hydrogenated product resulting from a consecutive reaction (Scheme 1); it is noted that the change in chemoselectivity is between an intermediate product (3-VA) and the final hydrogenation product (3-EA), rather than the more desirable switch between NO_2_ and C=C bond reduction.^19^ Du *et al.* reported another approach to change the chemoselectivity of a single atom Ru catalyst supported on TiO_2_, and they achieved this by altering the support from an amorphous porous ultrathin nanosheets of TiO_2_ to crystalline TiO_2_. Ru/amorphous TiO_2_ exhibited >99% selectivity for 3-VEA at conversion levels above 98%; in contrast, Ru/crystalline TiO_2_ showed 90% selectivity for 3-EA at 50% 3-NS conversion.^20^ However, selectivity data for Ru/crystalline TiO_2_ at higher 3-NS conversion levels were not presented, suggesting that this change in chemoselectivity may not be observed at higher 3-NS conversion levels. Furthermore, Beier *et al.* modified the selectivity of supported Pt catalysts by altering the pH of the reaction medium, achieved by homogeneous additives such as trifluoroacetic acid;^8^ however, from a green and sustainable chemistry perspective, additive-free reaction systems are highly desirable. Other strategies to tune chemoselectivity include the addition of a second metal, such as introducing Sn into a Pt-based catalyst, and using different hydrogen donors, such as hydrazine, formic acid, or sodium borohydride.^21^ These strategies are complex and therefore not viable for industrial applications, leaving an outstanding need for a method to switch chemoselectivity between –NO_2_ and C=C bond reduction in 3-NS. Delivering such a method, with a clear understanding of the nature of the active site, is highly valuable both industrially and academically.

Here, we report a support-induced chemoselectivity (SIC) switch between Pt/TiO_2_ and Pt/Nb_2_O_5_ catalysts during the hydrogenation of 3-NS. Characterization using STEM, XPS, CO-DRIFTS, XAS, *in situ* IR, and DFT calculations provides evidence that Pt–support interfacial sites contribute to the observed chemoselectivity of supported Pt catalysts. Although metal–support interfacial sites have been identified as active sites for various reactions (*e.g.*, CO oxidation, selective oxidation, reverse water-gas shift),^22–24^ this study presents the first demonstration of a potential support induced chemoselectivity tuning during the hydrogenation of 3-nitrostyrene.

## Materials and methods

### Catalyst synthesis

#### Impregnation method

Pt nanoparticles supported on various materials were synthesized using the wet-impregnation method as described in previous studies.^25^ For a typical preparation of 1 g of supported Pt catalyst, a precise volume of an aqueous chloroplatinic acid solution (20 mg_Pt_ mL^−1^ of H_2_PtCl_6_) and deionized water (DI water) were combined in a 50 mL single-neck round-bottom flask to form a 16 mL precursor solution. This solution was stirred at 1000 rpm with a magnetic stir bar and heated in an oil bath to 60 °C, and this temperature was maintained for 10 minutes to ensure thorough mixing. Next, the appropriate amount of support material was gradually added to the precursor solution while stirring to achieve a homogeneous mixture. Once the support material was fully added, the oil bath temperature was increased to 95 °C, and the mixture was continuously stirred at 1000 rpm for a further 16 h to allow complete evaporation of the water. The resulting dried sample was then ground using a mortar and pestle to ensure uniformity. This sample is referred to as the dried-only sample. The finely ground dried only sample was subsequently reduced under a constant flow of 5% H_2_/Ar (100 ml min^−1^) at 450 °C for 4 hours, with a heating ramp of 10 °C per minute. Our earlier study reports that this high temperature heat treatment removes almost all the chloride ions from the catalyst.^26^ The number before the catalyst label represents the wt% of Pt present in the catalyst.

#### Sol immobilization method

Supported Pt nanoparticles (0.2 wt% Pt/TiO_2_ and 0.2 wt% Pt/Nb_2_O_5_) were synthesised using a very well-established sol-immobilisation method.^27–29^ A 150 mL glass beaker containing a magnetic stir bar was placed on a hot plate equipped with magnetic stirring. The required volume of an aqueous H_2_PtCl_6_ solution was added to the beaker, corresponding to the desired Pt loading. Subsequently, 28 mL of deionised water was added, and the solution was stirred at 800 rpm for 3–5 minutes. A 10% (w/w) aqueous polyvinyl alcohol (PVA) solution was then added at a PVA : Pt weight ratio of 1 : 1, followed by stirring for 20–30 minutes to allow for effective stabilisation of the Pt precursor. Reduction of the Pt precursor was achieved by the rapid addition of a freshly prepared aqueous NaBH_4_ solution (NaBH_4_ : Pt molar ratio = 4 : 1). Since NaBH_4_ decomposes readily in water, the solution was freshly prepared and kept cold prior to use. To minimise decomposition, a vial of deionised water was cooled in an ice bath before preparing the NaBH_4_ solution. The reducing agent was then added swiftly to the reaction mixture, leading to the formation of stabilised colloidal Pt nanoparticles. The solution was stirred at 800 rpm for an additional 30 min. To promote electrostatic adsorption of Pt colloids onto the support surface, a few drops of H_2_SO_4_ were added to adjust the pH to mildly acidic conditions. An appropriate amount of TiO_2_ or Nb_2_O_5_ support was then gradually added to the beaker containing Pt colloids under constant stirring. The mixture was stirred for 40 minutes at 800 rpm to allow complete immobilisation of Pt nanoparticles onto the support. After complete immobilisation, the resulting solid catalyst was recovered by filtration and washed thoroughly with 2 L of deionised water to remove residual PVA and unreacted species and other water-soluble compounds. Previous studies have proven that almost all water-soluble compounds are removed during this washing step.^30–32^ The washed material was then dried overnight in an oven at 110 °C. The dried catalyst was ground to a fine, homogeneous powder using a mortar and pestle and used without any further treatment.

#### Colloidal Pt nanoparticles

A 150 mL glass beaker containing a magnetic stir bar was placed on a hot plate equipped with magnetic stirring. The required volume of an aqueous H_2_PtCl_6_ solution was added to the beaker, corresponding to the desired Pt loading. Subsequently, 28 mL of deionised water was added, and the solution was stirred at 800 rpm for 3–5 minutes. A 10% (w/w) aqueous polyvinyl alcohol (PVA) solution was then added at a PVA : Pt weight ratio of 1 : 1, followed by stirring for 20–30 minutes to allow for effective stabilisation of the Pt precursor. Reduction of the Pt precursor was achieved by the rapid addition of a freshly prepared aqueous NaBH_4_ solution (NaBH_4_ : Pt molar ratio = 4 : 1). Since NaBH_4_ decomposes readily in water, the solution was freshly prepared and kept cold prior to use. To minimise decomposition, a vial of deionised water was cooled in an ice bath before preparing the NaBH_4_ solution. The reducing agent was then added swiftly to the reaction mixture, leading to the formation of stabilised colloidal Pt nanoparticles. The solution was stirred at 800 rpm for an additional 30 min.

#### Chemoselective hydrogenation of 3-nitrostyrene

Liquid phase catalytic chemoselective hydrogenation was carried out in a 50 mL moderate pressure glass batch reactor (commercial name: Colaver® reactor). The reactor was charged with 3-NS (0.2 mL, 1.39 mmol, substrate), toluene (8 mL, solvent), *o*-xylene (0.1 mL, internal standard), and the required amount of the catalyst prior to each reaction. After sealing the reactor, the reaction mixture was purged three times with pure nitrogen and then three times with pure hydrogen. The reactor was then pressurized to 3 bar H_2_. The glass reactor, containing the reaction mixture, was immersed in an oil bath pre-heated to the reaction temperature of 40 °C. The reaction commenced when the reaction mixture was stirred at 800 rpm. After the desired reaction time, the stirring was stopped, and the reactor was placed in an ice bath to quench the reaction. After the reactor temperature dropped below 5 °C, the pressurized hydrogen was slowly released. The liquid reaction mixture was separated from the solid catalyst by centrifugation, and a small aliquot of the reaction mixture was taken for quantitative analysis by gas chromatography (GC). The analysis was conducted using a Varian 450GC gas chromatograph equipped with an Agilent CP-Sil 5CB boiling point column, an FID detector and a CP8400 Autosampler, employing N_2_ as the carrier gas. Conversion and selectivity values were calculated using the internal standard (*o*-xylene) and calibration plots. For the control experiments with individual substrates, 1.39 mmol of nitrobenzene and 1.39 mmol of styrene were used. For the control experiment with a mixture of substrates, a combination of 0.695 mmol of styrene and 0.695 mmol of nitrobenzene was utilized. All other conditions were identical to those used in the 3-NS hydrogenation reaction.

### GC-MS

Unknown product peaks were identified using GC-MS. Solutions were analysed using a Shimadzu 2010 Plus gas chromatograph equipped with an Agilent Technologies VF-WAXms column and QP2010 SE mass spectrometer. Products were compared to the NIST database and for compounds for which had no match, computed spectra were generated using NEIMS in Ubuntu 20.04.^5^ These computed mass spectra were added to a database and the similarity of spectra was determined using the Stein and Scott dot product function in a Python.^6^

### Catalyst characterization

#### Scanning transmission electron microscopy (STEM)

The metal nanoparticles size and morphology of the supported Pt catalysts were characterized by aberration corrected scanning transmission electron microscopy (AC-STEM) using a Thermo Fisher Titan STEM (G2 80-200) equipped with a Cs probe corrector (CEOS) and a high-angle annual dark-field (HAADF) detector, operating at 200 kV. The particle size distribution was obtained based on the particle projected area which was measured in HAADF-STEM images. The TEM samples were prepared by directly dispersing the catalyst powders onto a copper grid coated with an amorphous holey carbon film.

#### X-ray photoelectron spectroscopy (XPS)

Two systems were applied to perform XPS analyses. A Kratos Axis Ultra-delay link detector spectrometer with monochromatic Al Kα radiation (source power 140 W). An analyzer pass energy of 160 eV was used for general survey scans, and 40 eV for detailed scans of specific elements. The second instrument was a Thermo Scientific K-Alpha^+^ spectrometer, which also used micro focused monochromatic Al Kα radiation but at a lower source power of 72 W, with analyzer pass energies of 40 and 150 eV for analyses. The raw data was analyzed with computer-aided surface analysis for XPS (CasaXPS), using escape depth-corrected sensitivity factors supplied by the instrument manufacturers.

#### *In situ*-diffuse reflectance infrared Fourier transform spectroscopy (*in situ* DRIFTS)

*In situ* DRIFTS was performed in an Agilent Cary 600 FTIR spectrometer equipped with a liquid N_2_-cooled MCT detector. For this experiment, *ca.* 30 mg of catalyst sample was placed in the high-temperature *in situ* DRIFTS cell (HVC-DRM-5, Harrick Scientific, USA) fitted with ZnSe windows, an ohmic heating device, and a water-cooling jacket. The catalyst was first reduced using 4% H_2_/Ar while ramping to 120 °C. The sample was reduced at this temperature for 1 h and then purged with Ar for another hour. After the catalyst reduction, the temperature was dropped to 40 °C, at which the background spectrum (64 scans) was collected with a resolution of 4 cm^−1^. The sample was then treated with 3-nitrostyrene until saturation using a mixture of 3-NS and acetone. This liquid mixture was added to the solid catalyst in excess whilst it was inside the *in situ* DRIFTS cell. After that addition, the cell was heated at 65 °C for 40 min to remove acetone. After that, Ar was then flown to remove any physisorbed species and, subsequently, 4% H_2_ in Ar was introduced to perform the hydrogenation reaction of the adsorbed species for 90 min. Spectra were recorded throughout the reaction every 2 min. The gas outlet was monitored by mass spectrometry.

#### CO-DRIFTS

For the CO-DRIFTS analysis, supported Pt catalysts were loaded in a DRIFTS Tensor 27 spectrometer and purged with 30 ml min^−1^ N_2_ for 30 min to record a baseline. The sample was then exposed to 10% CO/Ar until complete saturation of the Pt sites. The system was then flushed with pure N_2_ until there was no presence of atmospheric CO before the spectra were recorded.

### CO chemisorption

CO chemisorption was performed using a Micromeritics AutoChem II 2920 instrument equipped with a thermal conductivity detector (TCD). The supported Pt catalysts samples were loaded into a quartz U-tube between two pieces of quartz wool and pre-treated in a 10% H_2_/Ar flow (50 mL min^−1^) for 1 h at 300 °C (10 °C min^−1^ ramp). After this time, the carrier gas was switched to pure Ar (50 mL min^−1^) whilst the temperature was held at 300 °C to purge the system of residual H_2_ for 1 h. The sample was subsequently cooled to 35 °C and the gas flow was switched to He (50 mL min^−1^) and the TCD baseline was allowed to stabilize. Pulses of 1%CO/He were injected until peak areas remained constant which was assumed to indicate saturation of surface sites on the Pt surface. The dispersion, particle size and metal surface area were calculated following literature procedures.^7^ The dispersion (*D*) stoichiometry of CO/Pt was assumed to be 1. Particle size was calculated using the equation *d* (nm) = 1.12/*D*. Platinum surface area was calculated according to the equation Surface area (m^2^ g_Pt_^−1^) = 249.12*D*.

#### Inductively coupled plasma mass spectroscopy (ICP-MS)

The total Pt content of the supported Pt catalysts was quantified *via* inductively coupled plasma mass spectrometry (ICP-MS). Accurately weighted (2 mg) supported Pt catalysts were dissolved in 10 mL of *aqua regia* for at least 12 h. The final solution was diluted to 50 mL with HPLC grade water in a volumetric flask. Further dilutions were made if required. The quantitative analyses were done on an Agilent 7900 ICP-MS equipped with I-AS auto-sampler. All calibrants were matrix-matched and measured against a five-point calibration using certified reference materials purchased from PerkinElmer and certified internal standards acquired from Agilent.

#### X-ray absorption fine structure spectroscopy (XAFS)

Extended X-ray absorption fine structure spectroscopy (EXAFS) measurements were performed at B18 beamline at Diamond Light Source at Pt LIII edge (11.56 keV) in fluorescence mode. The sample was prepared on self-supporting pellet, prepared using cellulose as a binder, and mounted on a rack. Data processing and analysis were done using Athena and Artemis software from the Demeter IFEFFIT package.^8^ The FEFF6 code was used to construct theoretical EXAFS signals that included single-scattering contributions from atomic shells through the nearest neighbours, using Pt and O as scatterers. The fit was performed using a *k*-range between 3 and 13 Å^−1^ and an *R* range between 1 and 3 Å. The amplitude reduction factor (*S*^2^_0_) was fixed at 0.86, and the Debye–Waller factor (DW) was locked at 0.003 for both samples.

#### Density functional theory simulations

Density functional theory (DFT) was used to analyze the atomic models of Pt/TiO_2_ and Pt/Nb_2_O_5_. The Pythonic Atomic Simulation Environment (ASE)^33^ was used to handle and manipulate the models, and the Fritz-Haber Institute *ab initio* materials simulations (FHI-aims)^34^ software package was used for energy calculations. The PBE exchange–correlation functional was used based on literature with the light (2020) basis set of FHI-aims.^35–37^ PBE functional is a very well used exchange–correlation approximation functional for trend identification in material study, including for TiO_2_ and Nb_2_O_5_ systems. The error margins for adsorption energy are typically under 0.2 eV with van der Waals correction. Furthermore, the Tkatchenko–Scheffler dispersion correction was applied to ensure accurate representation of van der Waals interactions.^38^ To neutralise the relativistic effects within the system, zero-order regular approximation (ZORA)^39^ scalar potential was employed, and dipole correction was applied to counteract any surface dipole arising in the system. The anatase phase of the TiO_2_ and the orthorhombic (T) Nb_2_O_5_ bulk were used as base structural models because orthorhombic Nb_2_O_5_ and P25 TiO_2_ (predominantly anatase) were used in the experiments. A converged *k*-grid sampling density of 0.05 Å^−1^ and 0.03 Å^−1^ (Fig. S10) was used to ensure energy accuracy of 0.01 eV for TiO_2_ and Nb_2_O_5_ surface, respectively. Structural optimisation search was conducted by relaxing the atomic positions of each geometry with the Broyden–Fletcher–Goldfarb–Shanno (BFGS) algorithm^17,18^ until the force on each atom was less than 0.01 eV Å^−1^.

Surfaces for the optimised crystals were constructed and tested for stability, using a 40 Å vacuum orthogonal to the surface plane to prevent self-interaction (Fig. S11). A 6-layer thick TiO_2_ slab and a 5-layers thick Nb_2_O_5_ slab were used with at least 50% relaxed layers ensuring surface energy convergence of less than 0.02 J m^−2^ (Fig. S12). The most stable surface for each material (101) for TiO_2_ and (110) for Nb_2_O_5_ seven miller ((001), (010), (011), (100), (101), (110) and (111)) after surface energy test (Fig. S13). The surface energy for (101) TiO_2_ is reported as 0.69 J m^−2^ in the literature,^19^ which is comparable to 0.93 J m^−2^ calculated here. The surface energy for T-(110) Nb_2_O_5_ is calculated to be 0.65 J m^−2^ while the literature^12^ reports 1.03 J m^−2^ for B-(110) Nb_2_O_5_. The difference of 0.38 J m^−2^ appears due to the phase difference of the reported crystal. The cell parameters of (101) TiO_2_ calculated in this study (*a* = 3.785 Å, *c* = 9.370 Å) are also found to be in very good agreement.^20^ Similarly, the lattice parameters for T-Nb_2_O_5_ are reported to vary from *a* = 6.175–6.321 Å and *c* = 3.813–3.930 Å in literature.^12,21^ The lattice parameters derived herein are within this proximity with *a* = 3.849 Å and *c* = 6.156 Å, thus validating our model.

Thereafter, a 13-atom Pt cluster was selected with considerations to the topological magic number and symmetry.^22^ The pyramidal shape of the particle was chosen to improve the interaction between the support and the nanoparticle. The Pt_13_ cluster was optimised in the gas phase prior to deposition and optimisation on the TiO_2_ and Nb_2_O_5_ surfaces (Fig. S14). Finally, the nitrostyrene was adsorbed on the Pt_13_/TiO_2_ and Pt_13_/Nb_2_O_5_ systems and the adsorption energy was calculated as:*E*_ads_ = *E*_(Pt_13_/support)+nts_ − *E*_(Pt_13_/support)_ − *E*_nts_,where *E*_(Pt_13_/support)+nts_ is the energy of the system where nitrostyrene is adsorbed on either catalytic materials, *E*_(Pt_13_/support)_ is the energy of the corresponding Pt_13_/TiO_2_ or Pt_13_/Nb_2_O_5_ catalytic material, and *E*_nts_ is the energy of the isolated nitrostyrene molecule.

The total density of states (DOS) and the atom-projected DOS (PDOS) for the stable catalyst complexes are presented in Fig. S15. The DOS and PDOS were calculated for the metal atoms directly under the Pt_13_ cluster, and for the interfacial atoms with Δ*q*_atom_ > 0.005*e* for Ti and Δ*q*_atom_ > 0.01*e* for Nb. The PDOS of Nb atoms show unoccupied p- and s-orbital states near the Fermi Level (*E*_F_), facilitating preferable adsorption *via* –C=C group by allowing the interaction of the π electrons of the –C=C group with the available orbitals. Therefore, adsorption *via* the alkene group is more stable over the Nb_2_O_5_ support. In contrast, the Ti cations exhibit the presence of d-orbital states near *E*_F_, allowing higher stability of adsorption *via* the –NO_2_ group over TiO_2_.

## Results and discussion

### Chemoselective hydrogenation of 3-nitrostyrene

The active sites for the chemoselective hydrogenation of 3-NS over supported Pt nanoparticles are the Pt–support interfacial sites.^19,25^ Traditionally, the chemoselectivity of these catalysts have always been tuned by changing the metal. We wanted to change the support sites, instead of the metallic sites, to tune the chemoselectivity of this catalyst. For this, Pt nanoparticles supported on different supports were synthesized using the wet-impregnation method and tested for the liquid phase hydrogenation of 3-NS at 40 °C and 3 bar H_2_ for 1 h. Detailed methodologies for catalyst synthesis, the hydrogenation reaction, catalyst characterisation and quantitative analyses of the reaction mixture are provided in the SI. Among all the catalysts tested, 1%Pt/TiO_2_, 1%Pt/C, 1%Pt/Nb_2_O_5_, and 1%Pt/MgO (the number denotes the wt% of Pt, details in SI) showed different chemoselectivity results, albeit at different conversion levels, while other catalysts produced a mixture of products (SI, Fig. S1). Specifically, 1%Pt/TiO_2_ exhibited over 99% selectivity for 3-VA (NO_2_ group reduction), whereas 1%Pt/C and 1%Pt/Nb_2_O_5_ showed over 90% selectivity for 3-ENB (C=C bond reduction). Additionally, 1%Pt/MgO exhibited approximately 75% selectivity for the intermediate product (*N*-(3-ethenylphenyl)hydroxylamine) at 100% conversion level (SI, Fig. S1). This preliminary data gave a qualitative indication that the selectivity can be tuned by changing the support. Based on this initial result, and the significance of the selectivity switch between NO_2_ and C=C reduction, Pt/TiO_2_ and Pt/Nb_2_O_5_ catalysts were selected for further detailed studies due to their contrasting selectivity. Although Pt/C and Pt/Nb_2_O_5_ showed similar selectivity for 3-ENB, Pt/Nb_2_O_5_ was chosen due to a higher conversion rate. An investigation of the effect of Pt loading on the catalytic activity and selectivity of Pt/TiO_2_ and Pt/Nb_2_O_5_ revealed that 0.2%Pt loading gave the highest selectivity towards 3-VA and 3-ENB respectively and produced the least amount of undesired products (SI, Fig. S2). In our earlier study we found that 0.2%Pt loading gave the most active catalyst for the Pt/TiO_2_ system.^25^ For these reasons 0.2%Pt loading was used for further studies. 1%Pt/TiO_2_ and 1%Nb_2_O_5_ catalysts also showed very high activity, probably because of more Pt surface area available for the catalytic reaction. However, this needs further investigation, and this aspect is beyond the scope of this article.

Fig. 1a and b compare the time online catalytic data of Pt/TiO_2_ and Pt/Nb_2_O_5_ for the hydrogenation of 3-NS at identical reaction conditions. The data clearly demonstrates that 0.2%Pt/TiO_2_ has exceptional selectivity for 3-VA (NO_2_ reduction, >99% selectivity at 88% conversion) whereas 0.2%Pt/Nb_2_O_5_ has high selectivity for 3-ENB (C=C reduction, *ca.* 80% selectivity at 50% conversion). Over longer reaction times, the selectivity for 3-ENB decreases, while the selectivity for 3-EA increases due to the consecutive hydrogenation reaction. Notably, the selectivity for 3-VA remains below 2% throughout the reaction with 0.2%Pt/Nb_2_O_5_. This shows that the two catalysts, 0.2%Pt/TiO_2_ and 0.2%Pt/Nb_2_O_5_, catalyse the complete hydrogenation of 3-NS to 3-EA *via* two distinct pathways: 0.2%Pt/TiO_2_ through 3-VA, and 0.2%Pt/Nb_2_O_5_ through 3-ENB. The simple support-induced chemoselectivity (SIC) is distinct from other chemoselective heterogeneous catalysts reported for this hydrogenation reaction. These results clearly suggest a support dependent chemoselectivity switch between 0.2%Pt/TiO_2_ and 0.2%Pt/Nb_2_O_5_.

**Fig. 1:**
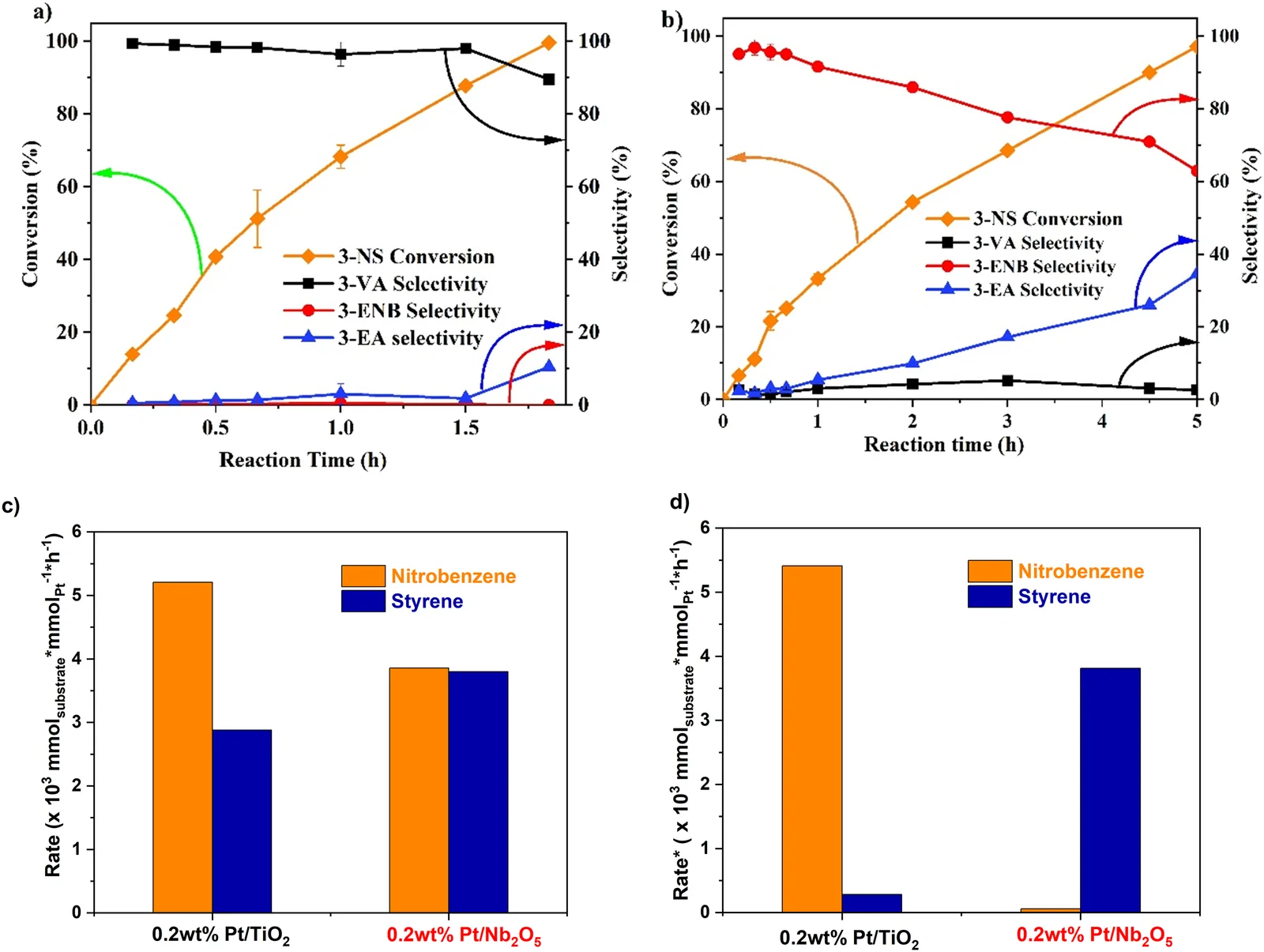
Catalytic performance of Pt/TiO_2_ and Pt/Nb_2_O_5_ catalysts for the hydrogenation of 3-nitrostyrene. The hydrogenation of 3-nitrostyrene (1.39 mmol) is performed over (a) 0.2%Pt/TiO_2_ (b) 0.2%Pt/Nb_2_O_5_. To further investigate reaction selectivity, (c) presents the separate hydrogenation of nitrobenzene (1.39 mmol) and styrene (1.39 mmol) over 0.2%Pt/TiO_2_ and 0.2%Pt/Nb_2_O_5_, with rate calculated from Fig. S3 and actual Pt loading calculated using ICP (Table S3); and (d) presents data for the hydrogenation of a mixture of nitrobenzene and styrene mixture (total amount: 1.39 mmol), in equal molar ratio, over the same Pt/TiO_2_ and Pt/Nb_2_O_5_ catalysts. Reaction conditions: 1.39 mmol reactants, 8 mL toluene, 50 mg catalyst, 3 bar H_2_, 40 °C; 3-VA is 3-vinlyaniline, 3-ENB is 1-ethyl-3-nitrobenzene, 3-EA is 3-ethylaniline and 3-NS is 3-nitrostyrene.

Several control experiments were conducted to further understand the role of each component of the supported Pt catalysts in the SIC switch. Hydrogenation of 3-NS over the bare supports (TiO_2_ and Nb_2_O_5_) did not result in any product after 24 h. To eliminate the influence of support properties, such as acidity, in this switch in selectivity, 3-NS hydrogenation was performed using a physical mixture of equal quantities of 0.2%Pt/TiO_2_ and pure Nb_2_O_5_, and another reaction with a physical mixture of 0.2%Pt/Nb_2_O_5_ and pure TiO_2_. The addition of the pure secondary support materials did not alter the chemoselectivity of the supported Pt catalyst (SI, Fig. S4), suggesting that intimate contact between Pt and the specific supports is crucial for the change in chemoselectivity. Another set of control experiments was conducted by hydrogenating nitrobenzene and styrene using the two supported Pt catalysts individually (Fig. 1c). 0.2%Pt/TiO_2_ reduces nitrobenzene at a higher rate (5207 mmol_nitrobenzene_ mmol_Pt_^−1^ h^−1^) compared to styrene (2879 mmol_styrene_ mmol_Pt_^−1^ h^−1^). However, the 0.2%Pt/Nb_2_O_5_ catalyst reduces both substrates at similar rates (3856 mmol_nitrobenzene_ mmol_Pt_^−1^ h^−1^ for nitrobenzene reduction and 3798 mmol_styrene_ mmol_Pt_^−1^ h^−1^ for styrene reduction). An equimolar mixture of nitrobenzene and styrene was hydrogenated using 0.2%Pt/TiO_2_ and 0.2%Pt/Nb_2_O_5_, and the results (Fig. 1d) show that 0.2%Pt/TiO_2_ selectively reduces nitrobenzene in the presence of styrene (5410 mmol_nitrobenzene_ mmol_Pt_^−1^ h^−1^*vs.* 284 mmol_styrene_ mmol_Pt_^−1^ h^−1^), while 0.2%Pt/Nb_2_O_5_ selectively reduces styrene in the presence of nitrobenzene (3812 mmol_styrene_ mmol_Pt_^−1^ h^−1^*vs.* 58 mmol_nitrobenzene_ mmol_Pt_^−1^ h^−1^). This chemoselective hydrogenation of a specific functional group in a mixture of substrates holds significant implications, particularly for the targeted hydrogenation of a single compound within a complex mixture. The results indicate that the observed switch in SIC when using 0.2%Pt/TiO_2_ or 0.2%Pt/Nb_2_O_5_ could be due to the preferential adsorption of one functional group over the other to the catalyst. A similar support induced chemoselectivity switch between 0.2%Pt/TiO_2_ and 0.2%Pt/Nb_2_O_5_ was also observed during the hydrogenation of 4-nitrophenylacetylene and 3-nitrophenylacetylene (SI, Tables S1 and S2).

### Electronic and geometric properties of 0.2%Pt/TiO_2_ and 0.2%Pt/Nb_2_O_5_

To understand the difference in SIC between 0.2%Pt/TiO_2_ and 0.2%Pt/Nb_2_O_5_, the electronic and structural properties of the catalysts were characterized using aberration corrected scanning transmission electron microscopy (AC-STEM) high-angle annular dark-field (HAADF) imaging, X-ray photoelectron spectroscopy (XPS), X-ray absorption spectroscopy (XAS), CO chemisorption and CO adsorbed diffuse reflectance infrared Fourier transform spectroscopy (CO-DRIFTS). The Pt loadings were quantitatively analyzed using inductively coupled plasma mass spectroscopy (ICP-MS), and the results (SI, Table S3) confirmed that the actual Pt loadings (0.196 wt% for Pt/TiO_2_ and 0.197 wt% Pt/Nb_2_O_5_) are in close agreement with the nominal loading (0.2 wt%).

Representative HAADF STEM images and particle size distribution data (obtained from *ca.* 250 particles) for 0.2%Pt/TiO_2_ and 0.2%Pt/Nb_2_O_5_ are given in Fig. 2(a) and (b), respectively. For both catalysts, most of the Pt particles were found to be very small (*ca.* 1 nm diameter), with a narrow particle size distribution for the peak population of ∼1 nm particles (FWHM is 0.4 nm). Notably, the 0.2%Pt/TiO_2_ catalyst exhibited larger particles (>2 nm) compared to the 0.2%Pt/Nb_2_O_5_ resulting in a larger mean particle size of 1.3 nm for 0.2%Pt/TiO_2_ compared to 1 nm for 0.2%Pt/Nb_2_O_5_. Complementary CO chemisorption experiments for the two catalysts, also suggested a larger apparent average Pt particle size for 0.2%Pt/TiO_2_ compared to 0.2%Pt/Nb_2_O_5_ but the technique suggested larger average Pt particle size (7.5 nm for 0.2%Pt/TiO_2_ and 1.8 nm for 0.2%Pt/Nb_2_O_5_ (SI, Table S4)). Consequently, the exposed Pt surface area for 0.2%Pt/Nb_2_O_5_ (154 m^2^ g_Pt_^−1^) is much higher compared to 0.2%Pt/TiO_2_ (37 m^2^ g_Pt_^−1^). The presence of SMSI in 0.2%Pt/TiO_2_ can result in this kind of Pt surface area difference. A major structural feature that is commonly observed in Pt/TiO_2_ catalyst system and to a lesser extent in Pt/Nb_2_O_5_ system is the partial or full coverage of Pt surface by partially reduced support material, widely known as strong metal support interaction (SMSI).^40,41^ STEM data of Pt/TiO_2_ did not show any evidence for the coverage of Pt nanoparticles by TiO_2_. However, we cannot rule out the presence of conventional SMSI in Pt/TiO_2_ sample based on this. The difference observed between these two techniques is likely due to the STEM sampling volume being relatively smaller than CO chemisorption, so it is more likely to miss large particles. The STEM images in Fig. 1(a) and (b) also reveal a higher density of small Pt particles on the surface of Nb_2_O_5_ compared to on TiO_2_ (with estimated densities of 0.07 and 0.01 particles per nm^2^ for 0.2%Pt/Nb_2_O_5_ and 0.2%Pt/TiO_2_, respectively). Given the identical 0.2 wt% Pt loading, the results are expected due to the larger mean Pt particle size for Pt/TiO_2_, as well as from the higher mean atomic number of the Nb_2_O_5_ support which provides a smaller specific surface area for the Pt to attach (even when the individual support particles are the same size). Though there is a difference in the mean Pt particle sizes between Pt/TiO_2_ and Pt/Nb_2_O_5_ catalysts, this variation may not be main reason for the observed chemoselectivity switch between the two catalysts. Two indirect evidence can used for this. One, in our previous study we reported that Pt/TiO_2_ with different mean particle sizes showed high selectivity for 3-VA (>95% selectivity) during the hydrogenation of 3-NS showing this high selectivity is independent of Pt particle size.^25^ Two, all Pt/TiO_2_ catalysts reported in Fig. S2 (SI), spanning six different Pt loadings (0.2%, 0.4%, 0.8%, 1.0%, 2.0%, and 5.0%), showed consistently high 3-VA selectivity (ranging from 81% to 97%) and consistently low 3-ENB selectivity (1–9%). Changing the Pt loading from 0.2% to 5.0% can safely be assumed to alter the Pt particle size, Pt coordination, Pt surface area, and fraction of interfacial Pt sites; indeed, Mirshekari *et al.* reported that increasing the Pt loading in a Pt/TiO_2_ catalyst leads to an increase in Pt particle size.^42^ Nevertheless, the catalytic data show that 3-VA remains the dominant product across all these catalysts, with 3-ENB selectivity staying below 10% throughout. The same trend holds for Pt/Nb_2_O_5_. Fig. S2 (SI) reports catalytic activities for Pt/Nb_2_O_5_ at the same six Pt loadings (0.2%, 0.4%, 0.8%, 1.0%, 2.0%, and 5.0%), all of which showed consistently high 3-ENB selectivity (77–91%) and consistently low 3-VA selectivity (3–9%). Again, although varying the Pt loading over this range is expected to change the Pt particle size, coordination, surface area, and fraction of interfacial sites, the data show that 3-ENB remains the dominant product across all these catalysts, with 3-VA selectivity staying well below 10% throughout. These results indicate that Pt particle size is not the primary factor responsible for the switch in chemoselectivity between the two catalysts. To further investigate the nanostructural differences between the two supported Pt catalysts, CO-DRIFTS was performed (Fig. 2c). Both 0.2%Pt/TiO_2_ and 0.2%Pt/Nb_2_O_5_ show CO vibrations at 2114 cm^−1^, 2087 cm^−1^ and 2067 cm^−1^. The 2087 cm^−1^ and 2067 cm^−1^ can be assigned to CO linearly adsorbed in an a-top geometry to metallic Pt^0^. These assignments are based on many previous reports on similar catalytic systems.^43–45^ The 2114 cm^−1^ can be assigned to CO adsorbed on electron deficient Pt sites, hence this could be either Pt single atoms on the support or Pt^*δ*+^ at the interfacial sites.^46,47^ However, the broad and asymmetric feature observed for 0.2%Pt/TiO_2_ indicates that this catalyst contains more heterogeneous Pt sites compared to the 0.2%Pt/Nb_2_O_5_ catalyst.^48^ The broad peak indicates a distribution of Pt electronic and coordination environments. Partial coverage or decoration of Pt particles by TiO_2−*x*_ and CO–CO dipole coupling (at higher CO coverage) could contribute to this broadening as well. However, more quantitative studies are needed to deconvolute these factors.^40,49^

**Fig. 2:**
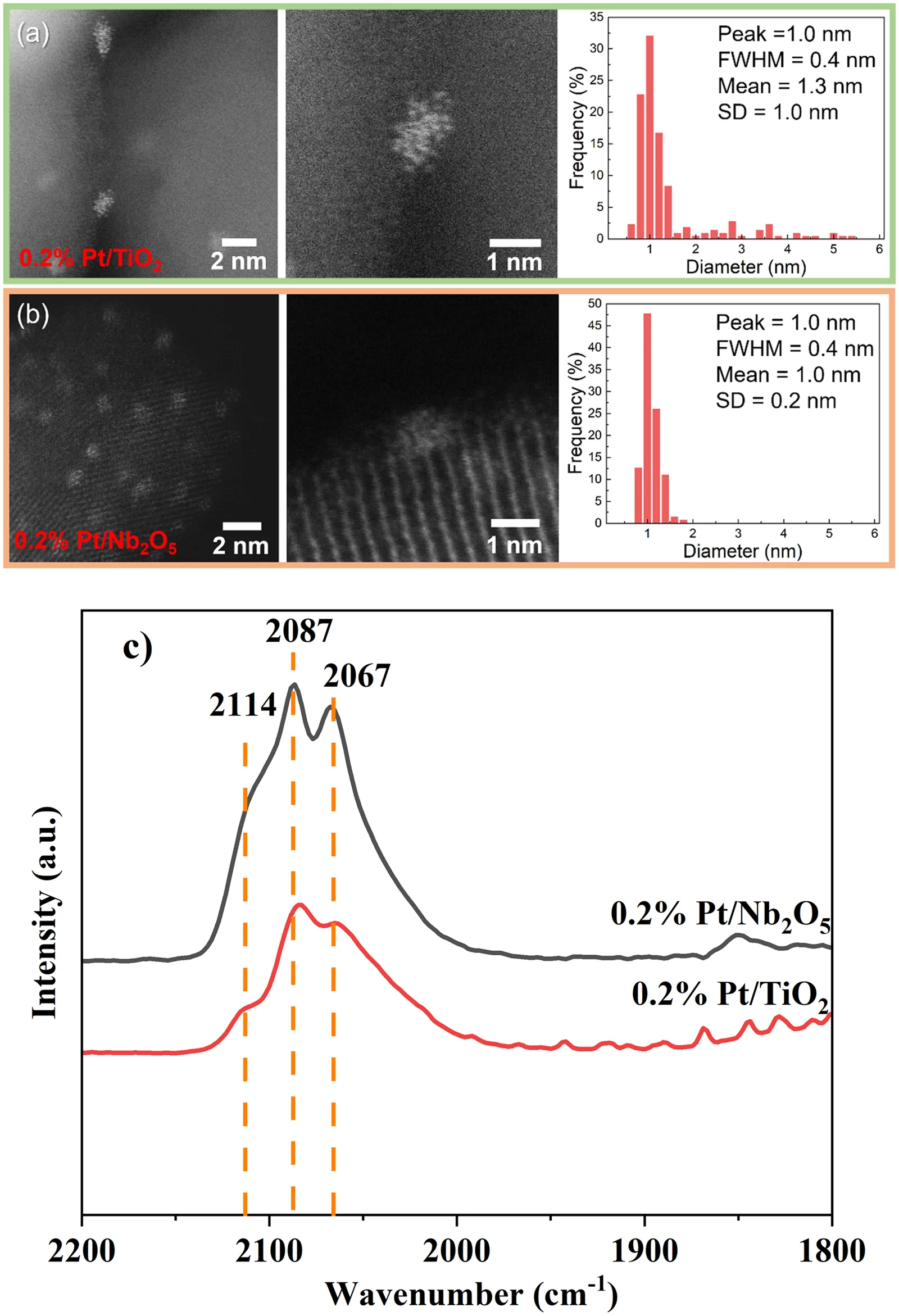
Representative STEM-HAADF images and particle size distribution histograms (obtained from 250 particles) of (a) 0.2%Pt/TiO_2_ and (b) 0.2%Pt/Nb_2_O_5_. (c) CO DRIFTS spectra of 0.2%Pt/TiO_2_ and 0.2%Pt/Nb_2_O_5_ (full spectra in SI (Fig. S5)).

The surface composition and oxidation states of Pt for both 0.2%Pt/TiO_2_ and 0.2%Pt/Nb_2_O_5_ were studied using X-ray photoelectron spectroscopy (XPS) as shown in Fig. 3. The 0.2%Pt/TiO_2_ catalyst exhibits a low Pt signal (Fig. 3(a)), suggesting poor dispersion or larger particle sizes in agreement with STEM and CO chemisorption. Whilst the Pt 4f_5/2_ peaks overlap with a Ti loss peak at *ca.* 75 eV, quantification has been achieved using the bare P25 as a model for the loss structure, together with fitting the Pt using line shapes derived from bulk materials. The Pt (4f_7/2_) peaks at 70.3 eV (0.2%Pt/TiO_2_)^25^ and 70.7 eV (0.2%Pt/Nb_2_O_5_)^50^ confirms the presence of Pt(0) on both catalysts; however, the TiO_2_-supported catalyst has a binding energy 0.4 eV lower than the Nb_2_O_5_-supported catalyst. Given bulk Pt has a binding energy of 71.1 eV, these shifts in binding energy may be attributed to particle-size effects, or an electronic effect from interaction with the support. It is noted, that the Nb_2_O_5_ supported catalyst also has some oxidic component at *ca.* 72 eV, which is lower than reported values for bulk PtO and PtO_2_,^35^ but again this may be due to particle size effects.

**Fig. 3:**
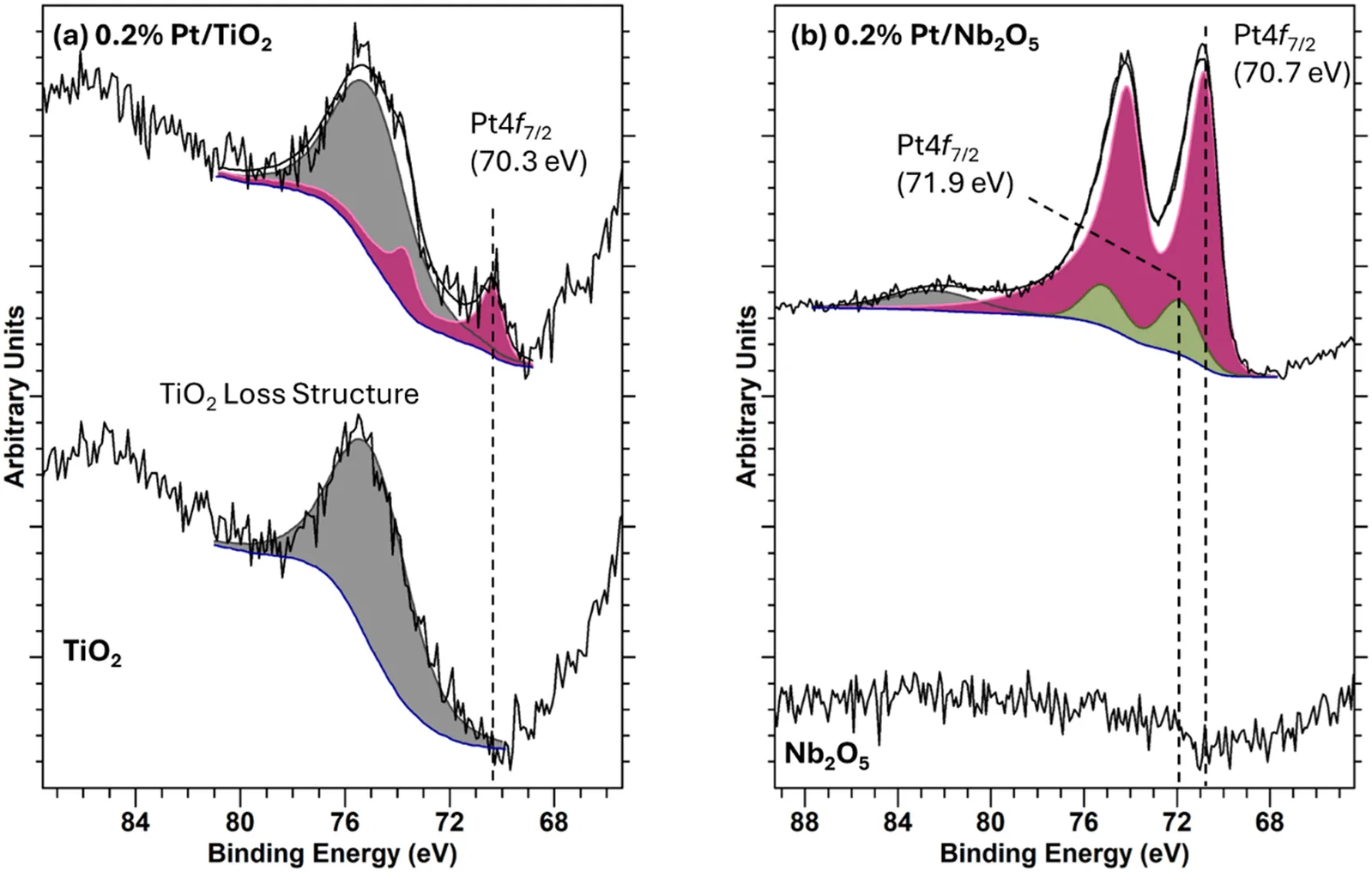
Pt 4f XPS spectra of (a) 0.2%Pt/TiO_2_ and (b) 0.2%Pt/Nb_2_O_5_.

To further understand the electronic structure of the Pt species on the two catalysts, they were characterized using X-ray absorption fine structure (XAFS) (Fig. 4). Different features are observed in the XANES for 0.2%Pt/TiO_2_ and 0.2%Pt/Nb_2_O_5_, and a linear combination fit using Pt, PtO and PtO_2_ (SI, Table S5) indicates that the Nb_2_O_5_-supported catalyst has a greater percentage of PtO_2_ when compared to the TiO_2_-supported catalyst. The EXAFS confirms the results of the XANES, with agreement that the Pt catalyst on Nb_2_O_5_ is more oxidized than the Pt catalyst on TiO_2_.

**Fig. 4:**
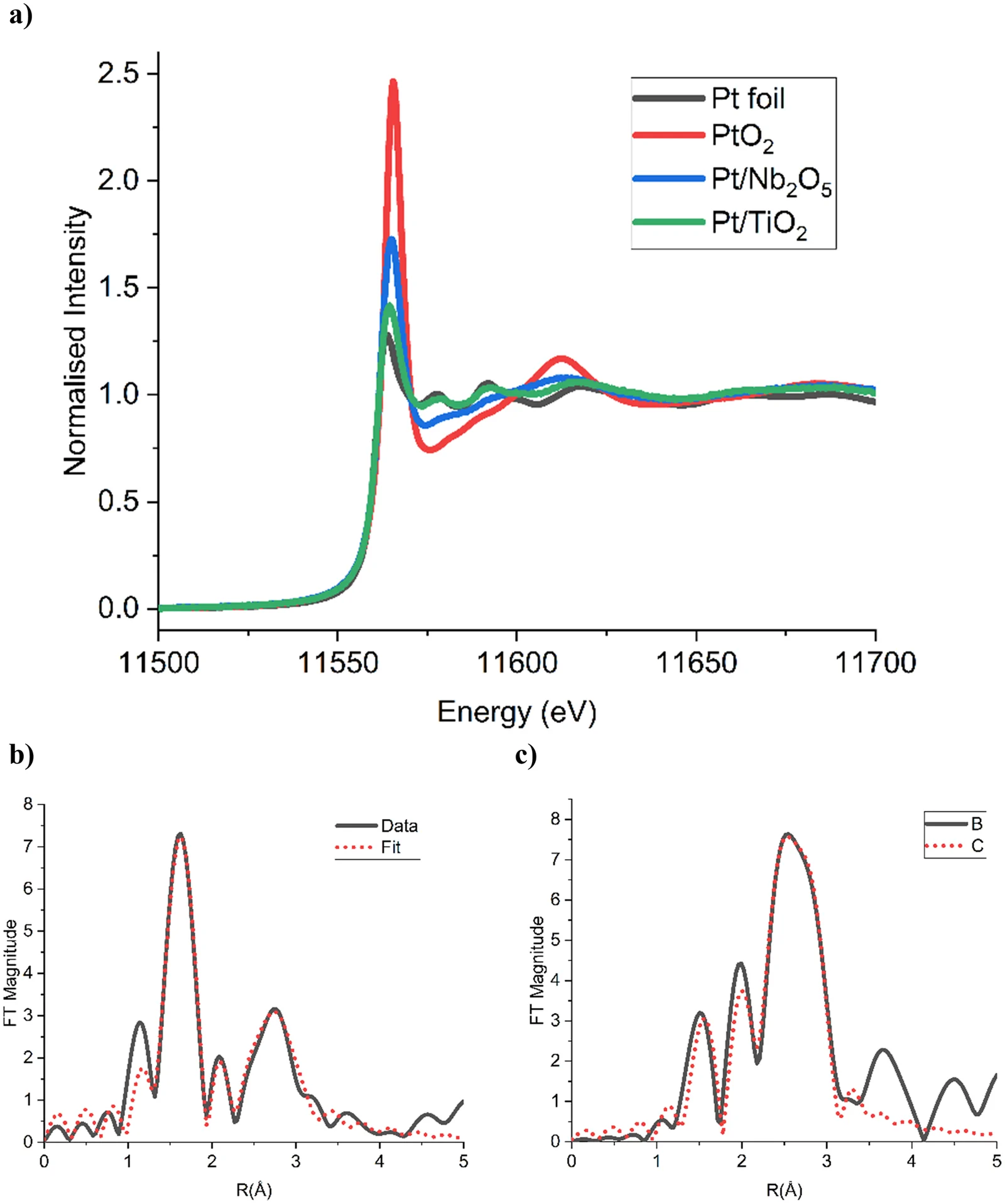
(a) Pt L_3_-edge XANES spectra of reference compounds compared to 0.2%Pt/Nb_2_O_5_ and 0.2%Pt/TiO_2_. (b) EXAFS-FT and fit (*k*^3^ weighting) of 0.2%Pt/Nb_2_O_5_ and (c) 0.2%Pt/TiO_2_. Results of the analysis are presented in the SI, Table S6. The corresponding *k*-plots are given in the SI (Fig. S6).

XAFS measurements were performed on the two catalysts, 0.2%Pt/TiO_2_ and 0.2%Pt/Nb_2_O_5_. The analysis of the XAFS data included interpretation of both XANES and EXAFS. The Pt L_3_-edge XANES spectrum is dominated by the dipole-allowed 2p_3/2_ to 5d_5/2_ transition of a core electron, which is known to be sensitive to the oxidation and coordination state of platinum. Fig. 4a shows the XANES plots, comparing the samples with Pt and PtO_2_ reference materials (SI, Fig. S7 for the FT spectra of these reference materials). The overview already reveals noticeable differences in the Pt species and environments present in these samples.

The XANES spectra in Fig. 4a show distinct differences between the two samples at the maximum in the normalised absorbance *μ*(*E*) trace, observed at ∼11 566 eV. For the 0.2%Pt/TiO_2_ sample, the maximum of the rising absorption edge is closer in intensity to the Pt foil reference than to PtO_2_. Additionally, features at 11 577 eV and 11 591 eV are common to both the foil and the 0.2%Pt/TiO_2_ sample, suggesting that metallic Pt is the predominant species, although some Pt oxide remains. The finding is consistent with the XPS results presented in Fig. 3. In contrast, the feature at 11 566 eV for the 0.2%Pt/Nb_2_O_5_ sample is more intense compared to both the foil and the 0.2%Pt/TiO_2_ sample. Along with weak features at 11 577 eV and 11 591 eV, a feature at 11 612 eV is also present in this data. The result indicates that the amount of metallic Pt in the 0.2%Pt/Nb_2_O_5_ sample is lower than in the 0.2%Pt/TiO_2_ sample, which is in agreement with the XPS analysis.

To verify and even extract further information from these samples, the EXAFS Fourier transform (FT) (phase uncorrected) data are plotted in Fig. 4b and c. For the 0.2%Pt/Nb_2_O_5_ sample, the spectrum exhibits a strong peak at ∼2.0 Å, characteristic of a Pt–O distance indicative of PtO_2_ species. The observation is further supported by a second peak at 2.75 Å in the FT, attributable to Pt–Pt scattering within this phase. Notably, these peaks are much lower in intensity compared to crystalline PtO_2_ due to structural disorder, indicating that the PtO_2_ species are dispersed. Additionally, there is a shoulder to the left of this peak at ∼2.50 Å, suggesting the presence of some metallic Pt. In contrast, the 0.2%Pt/TiO_2_ sample shows a broad peak with a maximum intensity at ∼2.63 Å, which is consistent with the expected response for Pt metal. Analysis of the EXAFS data for both samples, fitting Pt–O and Pt–Pt contributions, aligns with these empirical observations. The 0.2%Pt/Nb_2_O_5_ sample contains mainly oxidic Pt species, while the 0.2%Pt/TiO_2_ sample predominantly consists of metallic Pt species, which are either small in size and/or contain some oxidic Pt. Currently, we do not have direct evidence for the oxidation state of Pt under this reaction (reduction) condition. We believe that Pt reduces under the reaction (reduction) condition based on our previous *in situ* microfocus chemical computed tomography study and other *in situ* XANES studies on supported Pt catalysts.^51,52^ Hence, we believe that the oxidation state of Pt may not play a major role in this observed switching of chemoselectivity. However, further operando studies are needed to confirm this.

### *In situ* DRIFTS characterization of adsorbed species

*In situ* DRIFTS studies were conducted to investigate the role of adsorbed species in the SIC switch between 0.2%Pt/TiO_2_ and 0.2%Pt/Nb_2_O_5_ during the hydrogenation of 3-NS. Hydrogenation of 3-NS, nitrobenzene and styrene over 0.2%Pt/TiO_2_ and 0.2%Pt/Nb_2_O_5_ was performed in an *in situ* DRIFTS cell, and the results for 3-NS hydrogenation are presented in Fig. 5. Data for the hydrogenation of nitrobenzene and styrene are provided in the SI (Fig. S8). During the hydrogenation of 3-NS over the 0.2%Pt/TiO_2_ catalyst, the IR peaks at 1351 & 1533 cm^−1^, corresponding to the N–O stretching of the –NO_2_ group, rapidly decreased. Concurrently, an increase in the intensities of the IR peaks at 1494 and 1606 cm^−1^ were observed, attributed to the N–H stretching of the –NH_2_ group. The result indicates a higher rate of –NO_2_ hydrogenation for 0.2%Pt/TiO_2_ catalyst. After the complete hydrogenation of all the nitro groups (in 60 min), bands appear at 2962 cm^−1^ and 2933 cm^−1^, corresponding to the asymmetric stretching of the C–H bond in methyl (CH_3_) and methylene (CH_2_) groups, respectively. In contrast, the DRIFTS analysis of the same reaction over 0.2%Pt/Nb_2_O_5_ initially displays a rapid increase in the peaks at 2962 and 2933 cm^−1^, corresponding to hydrogenation of the C=C bond, while the peaks at 1351 and 1533 cm^−1^, corresponding to the NO_2_ group, were very slow to disappear. The results clearly show that NO_2_ group of 3-NS is preferentially hydrogenated over 0.2%Pt/TiO_2_, and the C=C bond is preferentially hydrogenated over 0.2%Pt/Nb_2_O_5_. These findings corroborate the observations from the catalytic experiments.

**Fig. 5:**
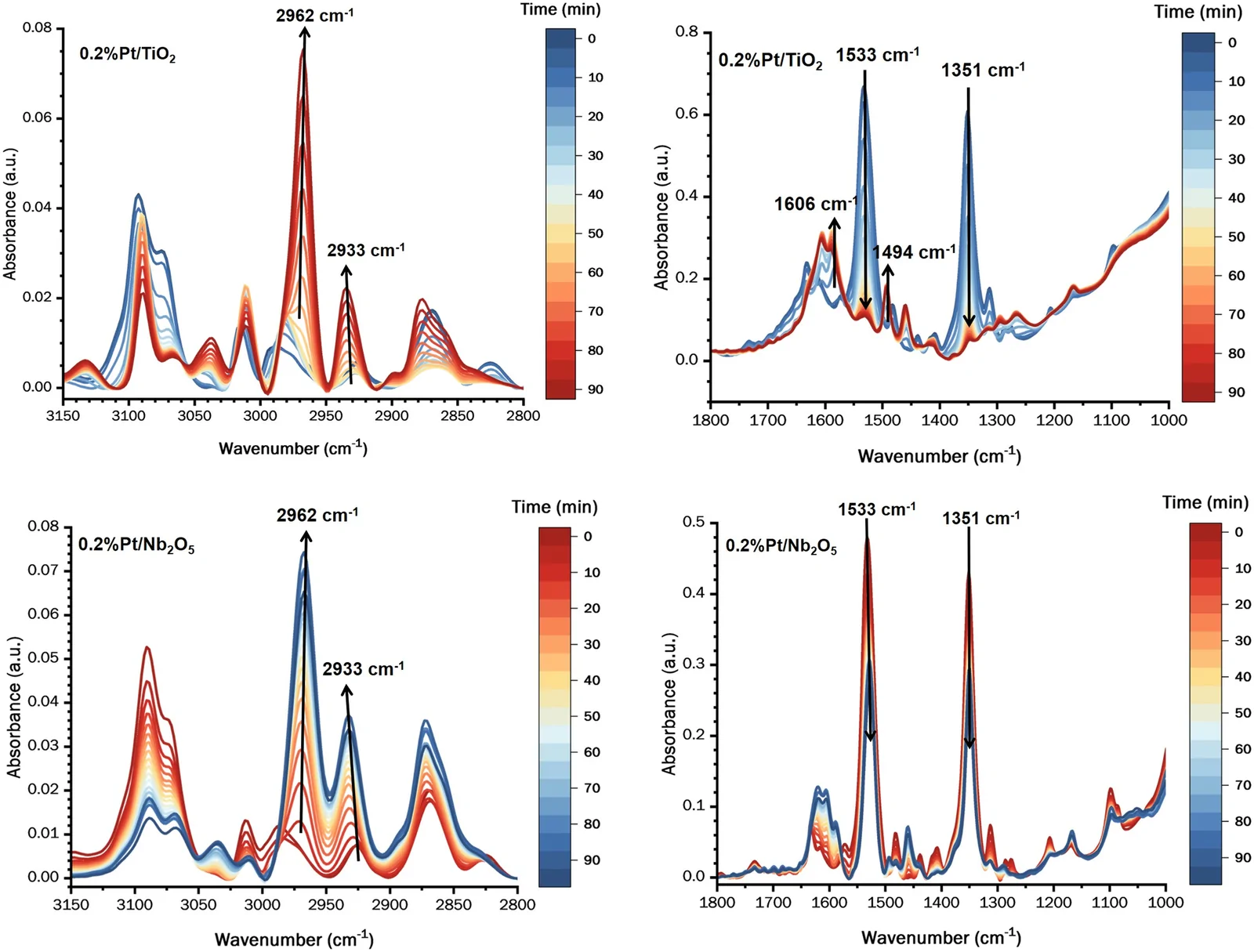
*In situ* DRIFTS spectra of 0.2%Pt/TiO_2_ (top) and 0.2%Pt/Nb_2_O_5_ (bottom) during the hydrogenation of 3-nitrostyrene at 40 °C.

Although the catalytic, spectroscopic, and control experiments demonstrate a support-dependent chemoselectivity switch, they do not uniquely identify the underlying mechanism. Several factors could contribute to the observed behaviour, including differences in Pt electronic structure, oxidation state, local metal–support interfacial structure, adsorption geometry, and support-mediated substrate interactions. To explore whether support-dependent adsorption preferences could contribute to the observed selectivity trends, DFT calculations were undertaken to provide qualitative insight.

### Density functional theory modelling of 3-NS adsorption on Pt/TiO_2_ and Pt/Nb_2_O_5_

Density functional theory (DFT) simulations were conducted to examine the interactions between Pt and the catalyst supports, as well as the interactions between the supported Pt catalysts and 3-NS.^36,37,53–55^ For this purpose, single atom Pt and a Pt_13_ cluster were each deposited on slab models of the support materials. Charge analysis of the reduced single atom Pt/TiO_2_ shows a marginally more negative charge (−0.20*e*) on Pt compared to the charge on Pt in pristine Pt/Nb_2_O_5_ (−0.18*e*), which aligns with experimental observations that Pt on TiO_2_ is more electron rich than on Nb_2_O_5_. A representative Pt_13_ cluster with pyramidal geometry was chosen for deposition on stable TiO_2_ and Nb_2_O_5_ surfaces to study 3-NS adsorption.^36,37,53–55^ While the experimentally observed catalysts exhibit distributions in particle size, dispersion, and oxidation state, the Pt_13_ cluster was employed as a computationally tractable model of the Pt–support interfacial environment rather than as an exact representation of the catalyst structure. Pt_13_ has been widely adopted in previous adsorption and reaction studies^56,57^ and offers a practical compromise between computational feasibility and practical relevance. Furthermore, Pt_13_ is known for its enhanced stability due to its closed-shell geometric configuration, as 13 is a magic number for Pt nanoclusters. The pyramidal geometry enhances the interaction between the base of the cluster and the support surface, resulting in a greater number of edge atoms compared to other geometric arrangements and thus aiding our analysis. These edge atoms serve as active sites for nitrostyrene adsorption; and, at the support surface, the change in charge of individual atoms after Pt_13_ cluster deposition (Δ*q*_atom_) is greatest at or near the metal–support interface, and Δ*q*_atom_ decreases with increasing distance from the Pt cluster (Fig. 6). The charge distribution for single Pt atom deposited on both the support surfaces shows a similar Δ*q*_atom_ trend (SI, Fig. S9), confirming that the charge distribution is a function of the Pt metal rather than of the cluster size or shape.

**Fig. 6:**
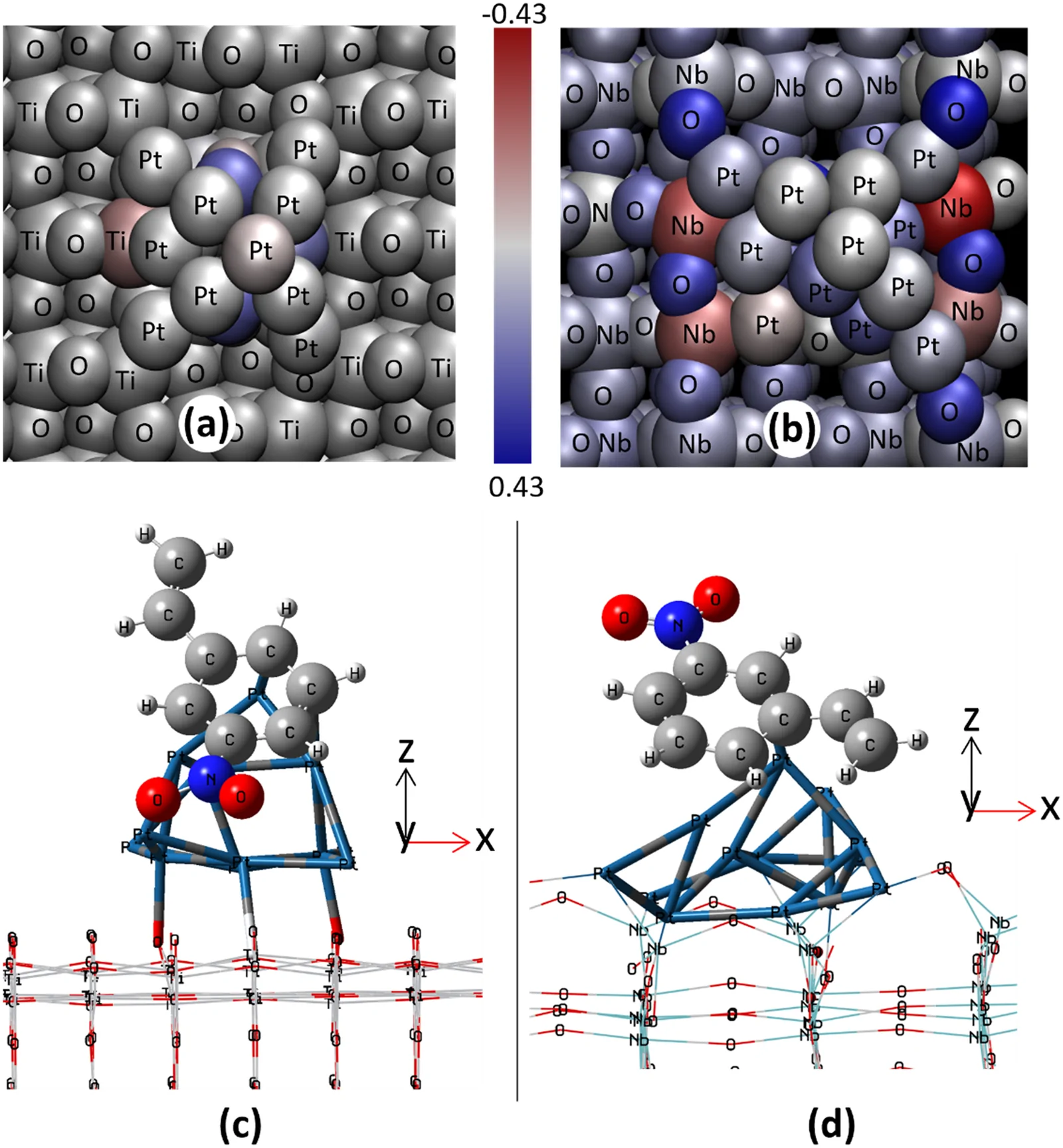
DFT calculated changes in charge distribution (Δ*q*_atom_) at support surfaces following the deposition of a Pt_13_ cluster on (a) TiO_2_, and (b) Nb_2_O_5_. A scale bar is given for the colour scheme, with red and blue representing net charge loss and accumulation, respectively; and DFT calculated 3-NS adsorption geometries (c) *via* NO_2_ group on Pt_13_/TiO_2_, and (d) *via* C=C group on Pt_13_/Nb_2_O_5_.

Interfacial sites are reported as the active site for catalytic reactions,^58^ and thus 3-NS was adsorbed with its functional groups interacting with the interfacial atoms of Pt_13_/TiO_2_ and Pt_13_/Nb_2_O_5_, where the most significant charge changes occur. Adsorption *via* the NO_2_ and C=C group were considered for both systems (*i.e.*, four configurations in total). Adsorption of 3-NS on Pt_13_/TiO_2_*via* the NO_2_ group (Fig. 6(c)) was 0.27 eV more stable than *via* the C=C group. In contrast, adsorption of 3-NS on Pt_13_/Nb_2_O_5_*via* the C=C group (Fig. 6(d)) was 0.16 eV more stable than *via* the NO_2_ group. The results suggest that 3-NS is preferentially adsorbed near the interfacial sites of TiO_2_- and Nb_2_O_5_-supported Pt catalysts by the NO_2_ and C=C functional groups, respectively. The differences in adsorption energy provide a qualitative indication that support-dependent adsorption preferences at the Pt–support interface may contribute to the selectivity trends observed experimentally. The atom-projected density of states (PDOS) further suggests that the preferential adsorption of the C=C functional group over Pt_13_/Nb_2_O_5_ may relate to the presence of the unoccupied s-orbitals near the Fermi level of the support cation (Nb) available at the interfacial site. While the calculated energy differences are relatively small, these results provide a qualitative model for the distinct adsorption behaviour of 3-NS on Pt_13_/TiO_2_ and Pt_13_/Nb_2_O_5_. Such adsorption preferences may facilitate further reduction by hydrogen spillover from the Pt cluster, contributing to chemo-selective hydrogenation.^59^ It is important to distinguish metal oxide-based supports such as TiO_2_ and Nb_2_O_5_ from carbon supports because Pt/C catalyst exhibits catalytic behaviour similar to that of Pt/Nb_2_O_5_. Carbon supports possesses physicochemical properties that differ substantially from those of TiO_2_ and Nb_2_O_5_, including a high surface area, an extended sp-hybridised covalent framework, and distinct electronic characteristics arising from their delocalised π-electron system. In addition, the structure of carbon supports is fundamentally different from that of TiO_2_ and Nb_2_O_5_, which are crystalline metal oxides with well-defined long-range order and ionic surface sites. In the context of this work, the cationic properties of the metal oxide support, which originate from the unoccupied valence orbitals, are not observed in covalent C supports; and thus, the arguments presented here are not expected to hold for Pt/C. The explanation involving unoccupied Nb-derived s states near the Fermi level is developed specifically for the Pt/Nb_2_O_5_ system, and may be transferable to other ionic supports, but not necessarily to Pt/C, despite its similar selectivity towards 3-ENB. The adsorption behaviour of 3-NS on Pt/C is likely governed by different metal–support interactions. Although these interactions may produce similar catalytic selectivity, they are expected to operate through a different underlying mechanism that needs further investigation.

### Blocking of support–metal interfacial sites using poly-vinyl alcohol

To further demonstrate the critical role of metal–support interfacial sites in governing chemoselectivity, colloidal Pt nanoparticles (unsupported) were synthesised using polyvinyl alcohol (PVA) as a stabilising ligand and tested for chemoselective hydrogenation. As shown in Table 1, the colloidal Pt nanoparticles, in the absence of any support, exhibited no chemoselectivity. Furthermore, when these PVA-stabilised Pt colloids were immobilised onto TiO_2_ and Nb_2_O_5_ supports and tested under identical conditions, they still showed no chemoselectivity (Table 1, sol immobilisation catalysts). This lack of chemoselectivity could be attributed to the blocking of Pt–support interfacial sites by the stabiliser ligands, rendering these critical sites inaccessible.^60^ Previous studies have reported that when PVA stabilised metal colloids were immobilised on to a metal-oxide based supports, these stabiliser ligands can partially hinder these metal–support interfacial sites. They have proven this *via* CO adsorption, suppression of H_2_ spillover, solid-state ^13^C-NMR and HRTEM-EELS.^61–63^ Based on extensive studies on this catalyst preparation method we can rule out the role of other potential impurities such as sulphur or sodium in tuning the chemoselectivity of this catalyst. These impurities are effectively removed during the washing steps. In contrast, catalysts prepared *via* conventional impregnation method displayed pronounced chemoselectivity (Table 1), along with a clear support-induced chemoselectivity switching effect between Pt/TiO_2_ and Pt/Nb_2_O_5_. This observation, together with the catalytic and spectroscopic results, suggests that the metal–support interfacial sites play a major role in tuning the chemoselectivity of supported Pt catalysts for 3-nitrostyrene hydrogenation. However, further careful studies are needed to rule out the influence of other parameters such as Pt dispersion, accessible Pt surface area, strong metal support interaction in this chemoselectivity switch.

**Table 1:** Role of metal–support interfacial sites during chemoselective hydrogenation of 3-NS over supported Pt nanoparticles

Catalyst	Synthesis method	Interfacial sites	Time (min)	3-NS conversion (%)	Products selectivity (%)		
					VA	ENB	EA
Colloidal Pt	No support	No interfacial sites	120	45	8	29	63
	(PVA stabilised)						
0.2%Pt/TiO_2_	Impregnation	Available	60	68	97	3	0
0.2%Pt/TiO_2_	Sol immobilization	Interfacial sites are blocked	120	61	56	19	26
	(PVA stabilised)						
0.2%Pt/Nb_2_O_5_	Impregnation	Available	120	54	4	86	10
0.2%Pt/Nb_2_O_5_^a^	Sol immobilization	Interfacial sites are blocked	120	44	15	61	24
	(PVA stabilised)						

a 1.58 mmol of 3-nitrostyrene. 3-VA is 3-vinlyaniline, 3-ENB is 1-ethyl-3-nitrobenzene, 3-EA is 3-ethylaniline and 3-NS is 3-nitrostyrene.

## Conclusion

During the hydrogenation of 3-nitrostyrene over supported Pt catalysts, a support-induced switch in the reaction chemo selectivity was observed for 0.2%/TiO_2_ and 0.2%/Nb_2_O_5_. Catalyst testing and control experiments with nitrobenzene and styrene demonstrate that 0.2%Pt/TiO_2_ selectively reduces the NO_2_ group to form 3-vinyl aniline, whereas 0.2%Pt/Nb_2_O_5_ selectively reduces the C=C bond to form 3-ethylnitrobenzene. Advanced characterization and *ab initio* calculations rationalize the contrasting selectivities between the two supported Pt catalysts; the selectivity differences correlate with the differences in the Pt–support interfacial environment, which change the chemoselectivity of these catalysts during the hydrogenation of 3-nitrostyrene. DFT calculations suggest that the presence of unoccupied states near the Fermi level of interfacial Nb species may favour adsorption *via* the C=C functional group, providing an explanation for the observed selectivity. While this model explains the behaviour of Pt/Nb_2_O_5_, it is not expected to directly extend to carbon supports, which possess a covalent sp-hybridised electronic structure with delocalised π-electrons rather than the unoccupied cation states present in metal oxides. The similar selectivity observed for Pt/C therefore suggests that analogous catalytic outcomes may arise from different metal–support interactions and adsorption mechanisms. When these Pt–support interfacial sites are blocked by stabilizer ligands (PVA), the catalysts exhibit no chemoselectivity. The catalytic results of two supported Pt catalysts, along with advanced characterization, demonstrate that the chemoselectivity of 3-NS hydrogenation can also be tuned by tailoring the Pt–support interfacial sites, by changing the support, rather than by changing the metallic sites, which provides an additional approach to design new efficient catalysts for chemoselective hydrogenation.

## Author contributions

LX performed the catalyst preparation and catalyst testing under the supervision of MS and SHT. MC performed CO-DRIFTS and GC-MS studies. KA performed the electronic structure calculations under the supervision of AJL. RC carried out electron microscopy studies of the catalyst under the supervision of SJH. DJM performed the XPS characterization of the catalysts. SMM and AMB analysed the X-ray absorption spectroscopy and *in situ* IR data. RQ suggested some experiments and supported LX in interpretations. MS directed the project. All authors contributed to the data analysis and drafting of this manuscript.

## Conflicts of interest

The authors declare no competing interests.

## Supplementary Material

CY-016-D6CY00493H-s001

## Data Availability

The data generated during this study are included in this paper and additional data can be found in the supplementary information (SI). The additional data include catalytic testing of Pt nanoparticles supported on different supports, effect of Pt loading, effect of adding a second support, chemoselective hydrogenation of 4-nitrophenyl acetylene and 3-nitro phenyl acetylene over Pt/TiO_2_ and Pt/Nb_2_O_5_ catalysts, ICP data, CO chemisorption data. Further data include isolated EXAFS and fit data of Pt/Nb_2_O_5_ and Pt/TiO_2_, results of linear combination fit analysis, and *in situ* DRIFTS spectra along with the computational data. The raw data for the results presented in this article can be found in the Cardiff University data catalogue. The 3-dimensional structures evaluated using the density functional theory method are uploaded to the NOMAD repository and can be accessed using the upload id Z5_ECjegSLSUPFd2DYzzrw. Supplementary information is available. See DOI: https://doi.org/10.1039/d6cy00493h.
