# Supplementary material for: Controlling chemoselectivity of heterogeneous Pt catalysts via a support induced effect
Source: Catal Sci Technol. 2026 Sep 29;16(22):7603–17. doi: 10.1039/d6cy00493h (PMC13647151; doi:10.1039/d6cy00493h)
Supplement: Supplementary file 2 [file CY-016-D6CY00493H-s001.pdf]

Supplementary Information (SI) for Catalysis Science & Technology.
This journal is © The Royal Society of Chemistry

Supporting information

Controlling Chemoselectivity of Heterogeneous Pt catalysts via a Support Induced Effect

Lifeng Xiao,[a] Kushagra Agrawal,[a] Rongsheng Cai,[b] Ruiyang Qu,[c, d] Sofia Mediavilla Madrigal,[d,e] Matthew Conway,[a] David J Morgan,[a] Stuart H Taylor,[a] Andrew M Beale,[e,f] Sarah J Haigh,[b] Andrew J. Logsdail,[a] Meenakshisundaram Sankar[a]*

a. *Cardiff Catalysis Institute, School of Chemistry, Translational Research Hub, Maindy Road, Cardiff University, Cardiff CF24 4HQ.*

b. *Department of Materials, University of Manchester, Manchester, M13 9PL UK.*

c. *Leibniz-Institut für Katalyse e.V. Albert-Einstein-Straße 29a 18059 Rostock Germany.*

d. *CHN Energy Europe Research GmbH; Englische Str. 21, Berlin, 10587, Germany.*

e. *Chemistry Department, University College of London, 20 Gordon Street, London, WC1H 0AJ UK.*

f. *Research Complex at Harwell, Rutherford Appleton Laboratory, Didcot, OX11 0FA UK.*

Corresponding author: Dr. M. Sankar, Cardiff Catalysis Institute, School of Chemistry, Translational Research Hub, Maindy Road, Cardiff University, Cardiff CF24 4HQ. Email: Sankar@cardiff.ac.uk

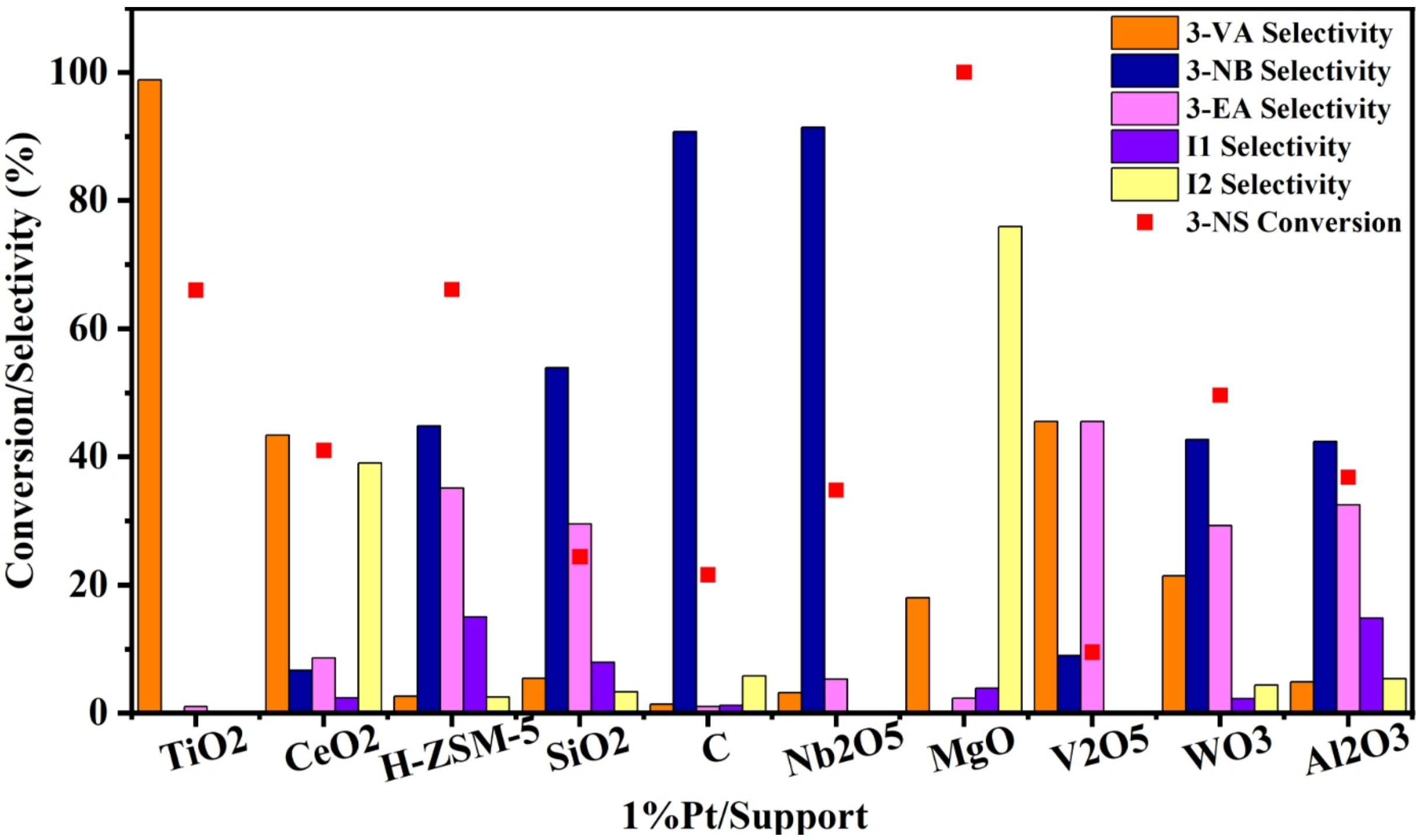

Figure S1: Effect of support on the catalytic performance of 1%Pt/Support catalyst for 3-nitrostyrene hydrogenation. Reaction condition: 3-NS: 1.39 mmol; toluene: 8 mL, catalyst :50 mg; H_2: 3 bar; Temperature: 40 °C; Time: 1 h. 3-NS: 3-nitrostyrene; 3-VA:3-vinlyaniline; 3-ENB: 1-ethyl-3-nitrobenzene, and 3-EA :3-ethylaniline, I1: 3-vinylnitrosobenzene I2: N-(3-ethenylphenyl)hydroxylamine and 3-NS is 3-nitrostyrene.

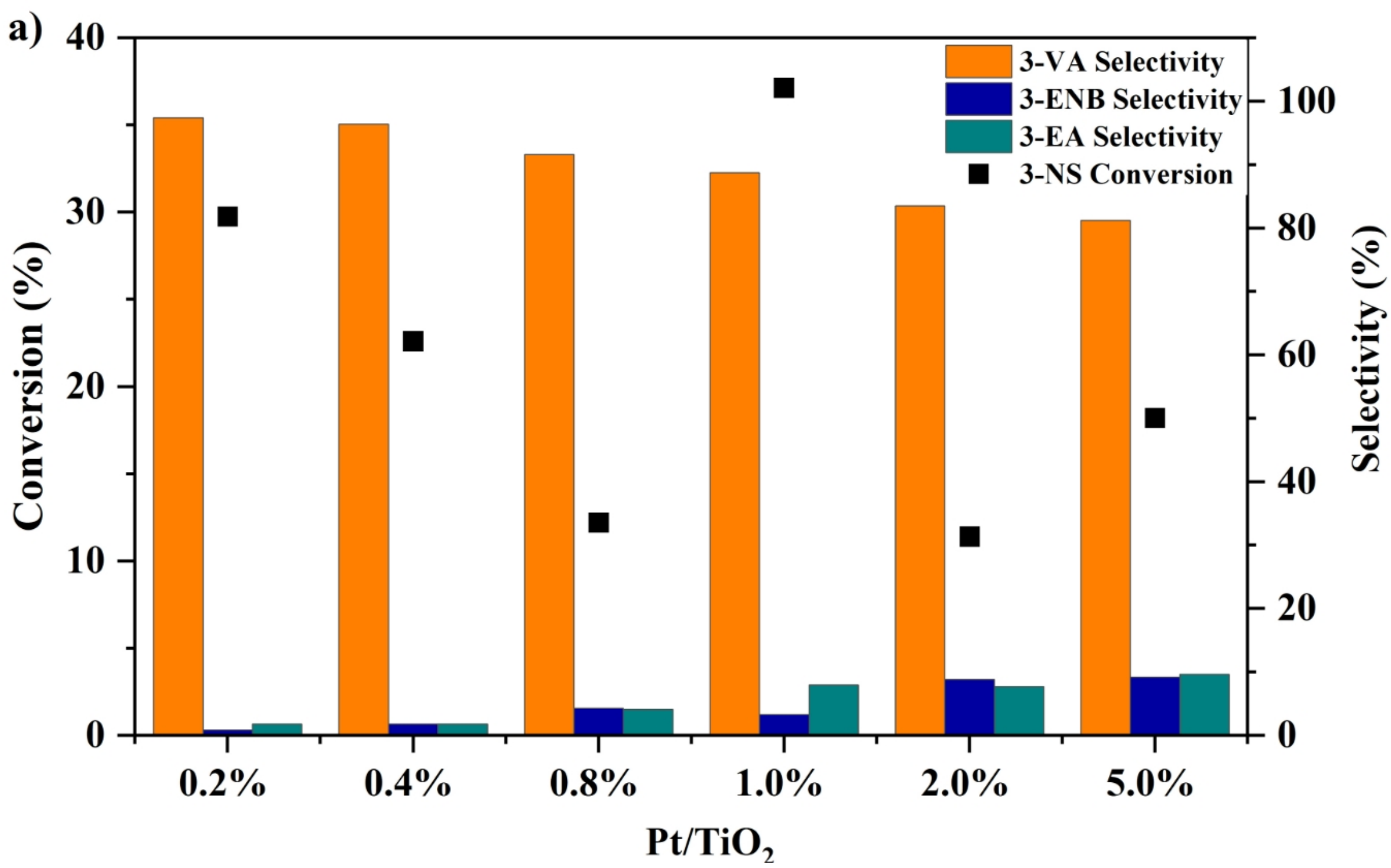
a)
3-VA Selectivity
3-ENB Selectivity
3-EA Selectivity
3-NS Conversion
Conversion (%)
Selectivity (%)
0
10
20
30
40
0
20
40
60
80
100
0.2%
0.4%
0.8%
1.0%
2.0%
5.0%
Pt/TiO2

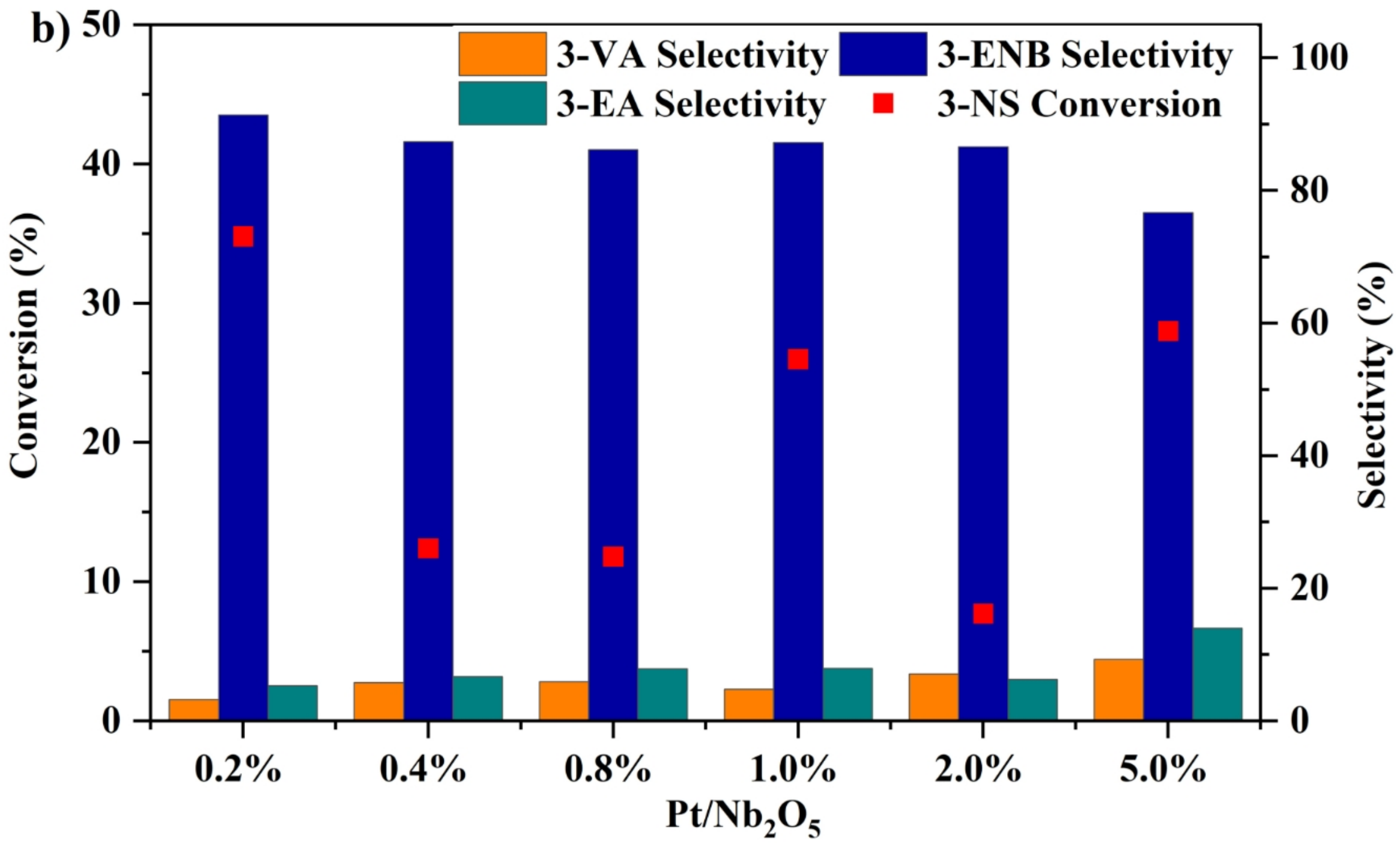

Figure S2: Effect of Pt loading (in wt.%) on the catalytic properties of (a) Pt/TiO_2 and (b) Pt/Nb_2O_5 during 3-nitrostyrene hydrogenation. Reaction conditions: 3-NS: 1.39 mmol; toluene: 8 mL; H_2: 3 bar; temperature: 40 °C; catalyst: 50 mg catalyst (for 0.2%Pt/TiO_2, 0.4%Pt/TiO_2, 0.8%Pt/TiO_2, 1% Pt/TiO_2), 25mg of 2% Pt/TiO_2 and 10mg of 5% Pt/TiO_2. 3-VA: 3-Vinlyaniline; 3-ENB: 1-Ethyl-3-nitrobenzene; 3-EA: 3-ethylaniline and 3-NS :3-nitrostyrene.

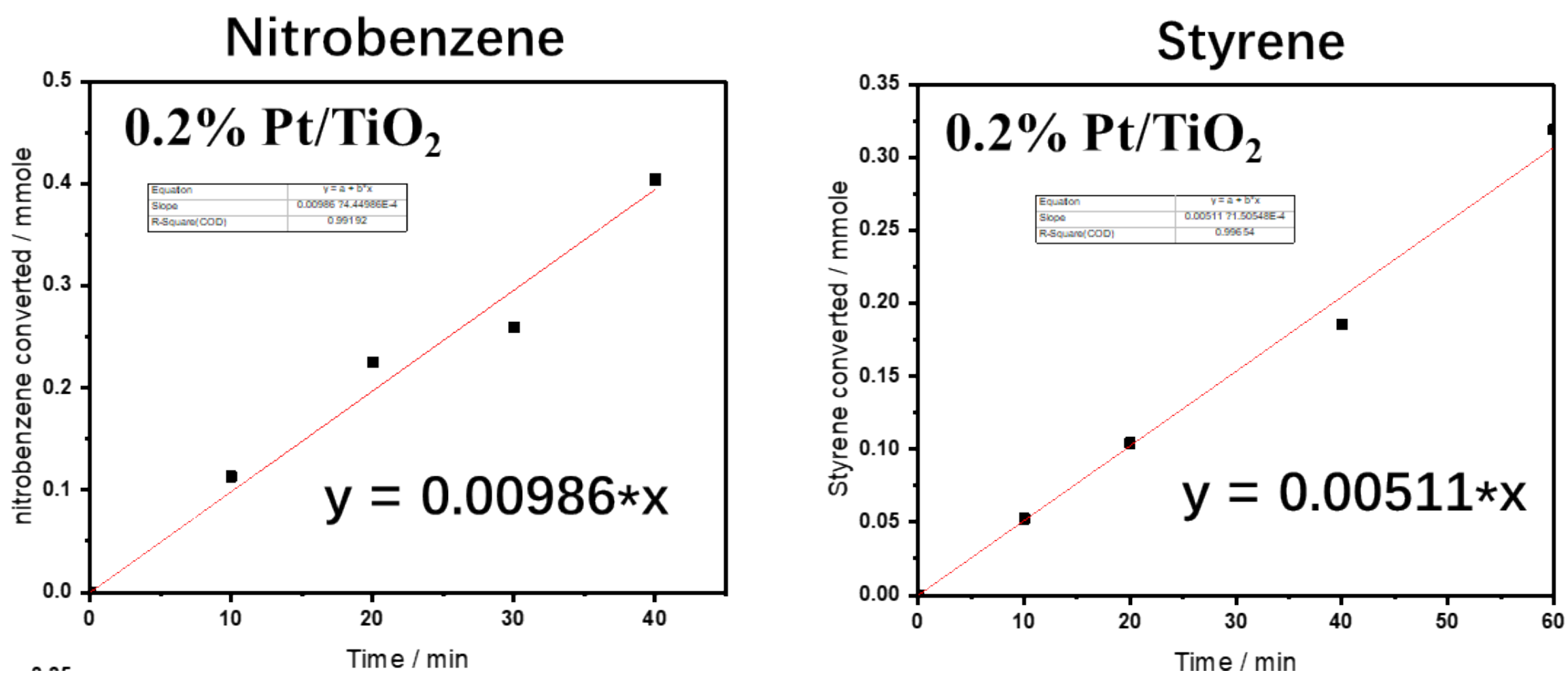

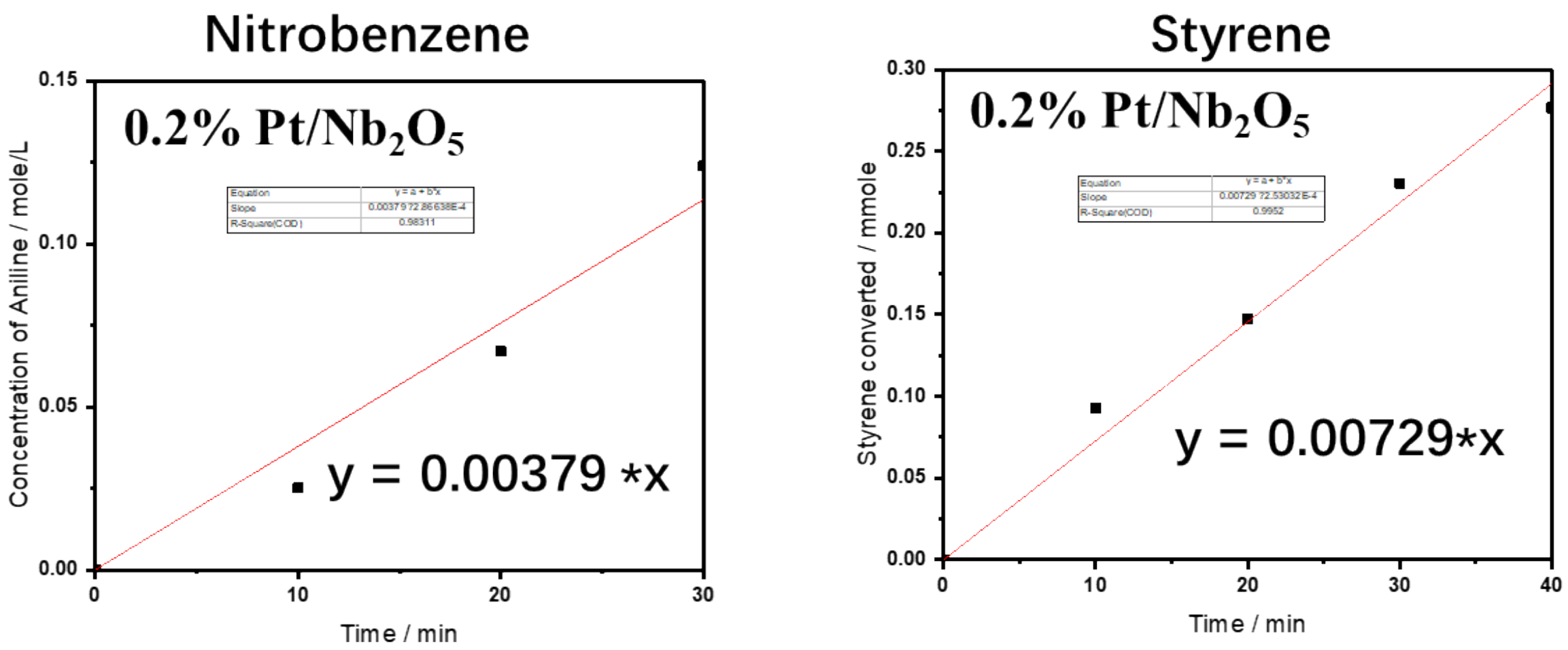

Figure S3. Initial rate data calculation for the control experiments using nitrobenzene and styrene individually over 0.2%Pt/TiO_2 and 0.2% Pt/Nb_2O_5. The initial rate calculation was done at below 20% substrate conversion. Reaction conditions: nitrobenzene or styrene : 1.39 mmol; toluene: 8 mL; catalyst :10 mg; H_2: 3 bar; Temperature: 40 ℃

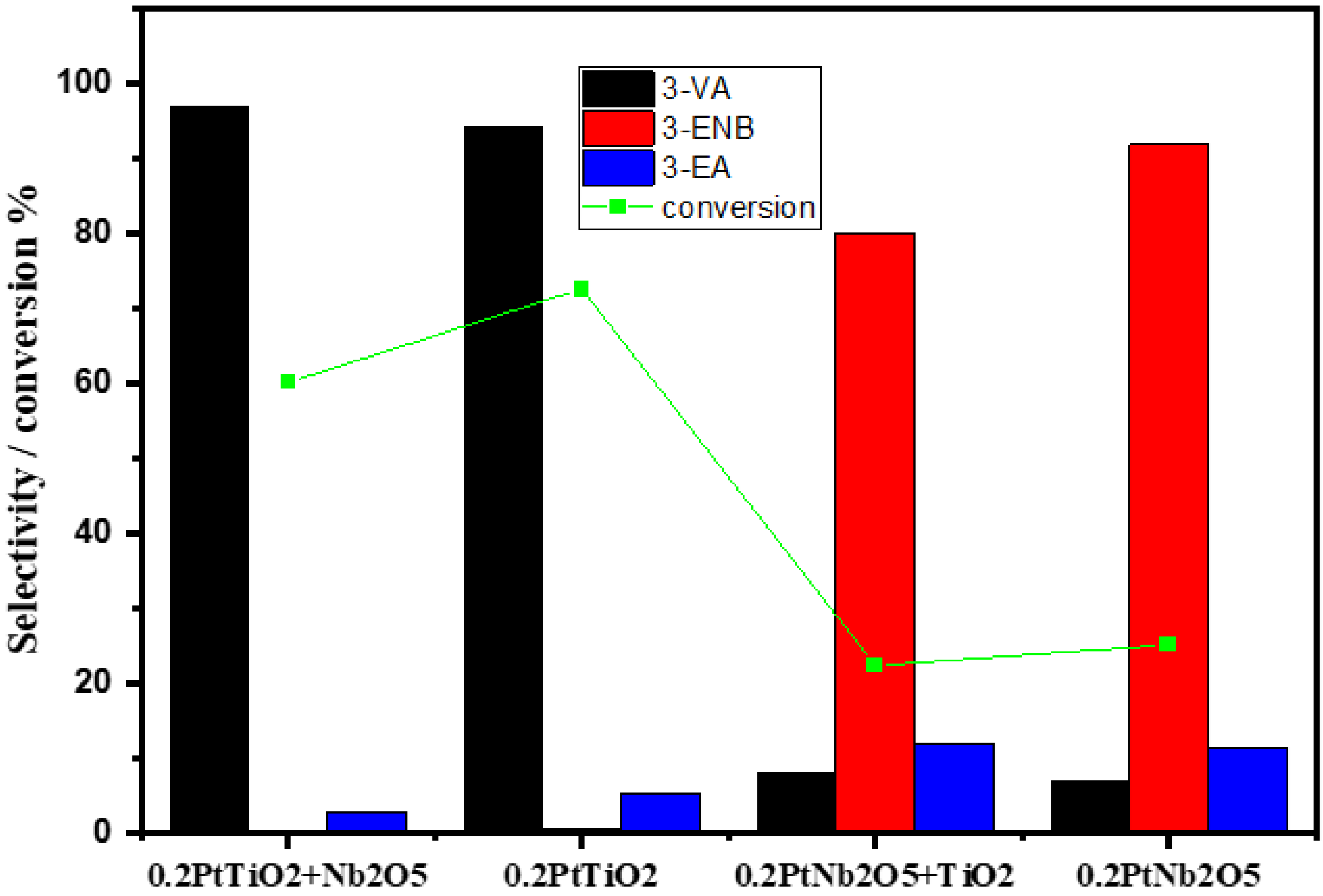

Figure S4. Effect of adding second support to 0.2%Pt/support as a physical mixture on the catalytic activity during 3-nitrostyrene hydrogenation. Reaction conditions: 3-NS: 1.39 mmol; toluene: 8 mL, catalyst :50 mg; additional support : 50 mg; H_2: 3 bar; Temperature: 40 °C; Time: 1 h.

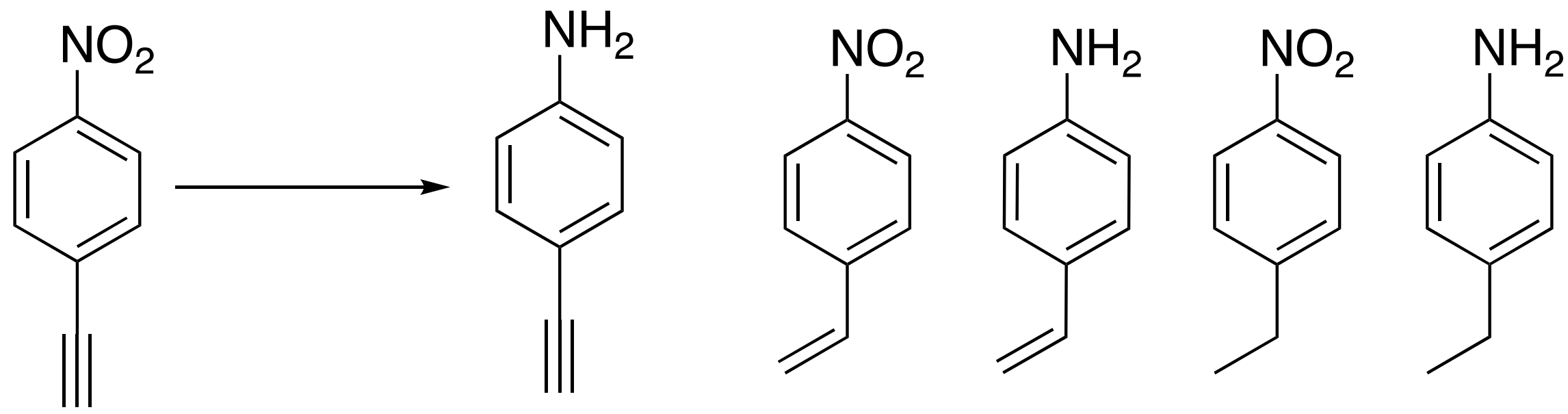

A B C D E

Table S1. Chemoselective hydrogenation of 4-nitrophenylacetylene over supported Pt nanoparticles.

Catalyst	Time (h)	Conversion (%)	Product selectivity (%)				
			A	B	C	D	E
0.2%Pt/TiO_2[a]	2	54	93	2.5	4.3	0.4	0
0.2%Pt/Nb_2O_5[b]	3.5	70	16	57	0	17	10

Reaction conditions: 4-nitrophenylacetylene (substrate): 1.38 mmol; o-xylene (internal standard) :0.1 mL; toluene (solvent): 8 mL; H_2 : 3 bar; temperature: 40 °C. [a] 50 mg catalyst, [b] 200 mg catalyst.

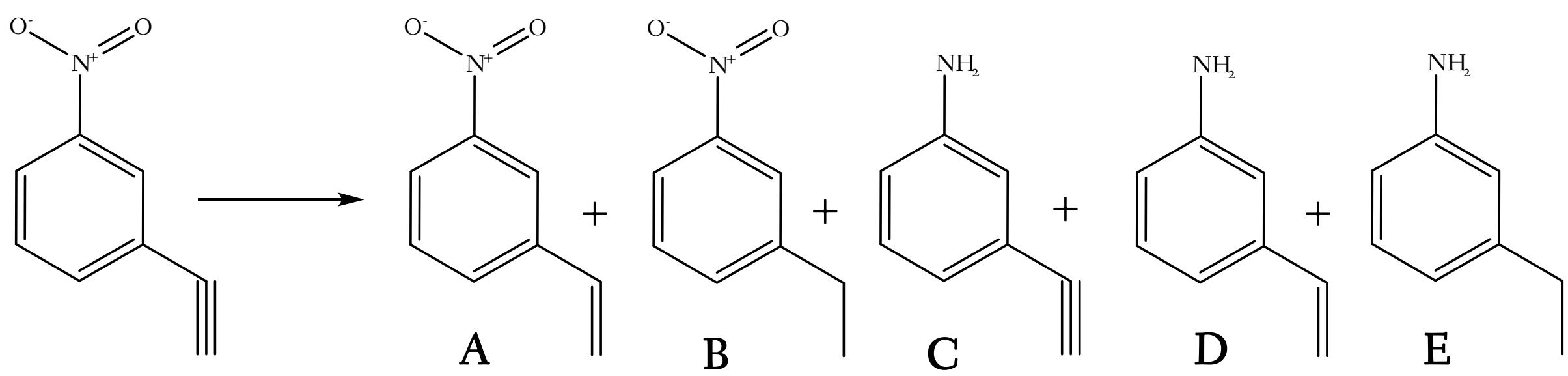

Table S2. Chemoselective hydrogenation of 3-nitrophenylacetylene over supported Pt nanoparticles.

Catalyst	Time (h)	Conversion (%)	Product selectivity (%)				
			A	B	C	D	E
0.2%Pt/TiO_2[a]	0.5	10	5	0	95	0	0
0.2%Pt/Nb_2O_5[b]	1.0	53	76	13	6	2	3

Reaction conditions: 3-nitrophenylacetylene (substrate): 1.58 mmol; o-xylene (internal standard) : 0.1 mL; toluene (solvent): 8 mL; H_2 : 3 bar; temperature: 40 °C. [a] 50 mg catalyst, [b] 200 mg catalyst.

Table S3. Quantitative analyses of Pt content of the catalysts using Inductively Coupled Plasma Mass Spectrometry (ICP-MS)

Sample name	Theoretical concentration expected mgPt/mL	Pt concentration obtained from ICP mgPt/mL
0.2% Pt/TiO_2	2	1.960
0.2% Pt/Nb_2O_5	2	1.966

*Table S4. CO Chemisorption results for the supported Pt catalysts**

Sample name	CO adsorbed (mmol g^{-1})	Metal dispersion (%)	Pt surface area ($m^2 g_{Pt}^{-1}$)	Particle Diameter (nm)
0.2 % Pt/Nb_2O_5	0.00640	62.4	154	1.8
0.2% Pt/TiO_2	0.00155	15.1	37	7.5

** Mass of metal = 0.2 wt%; stoichiometry factor = 1*

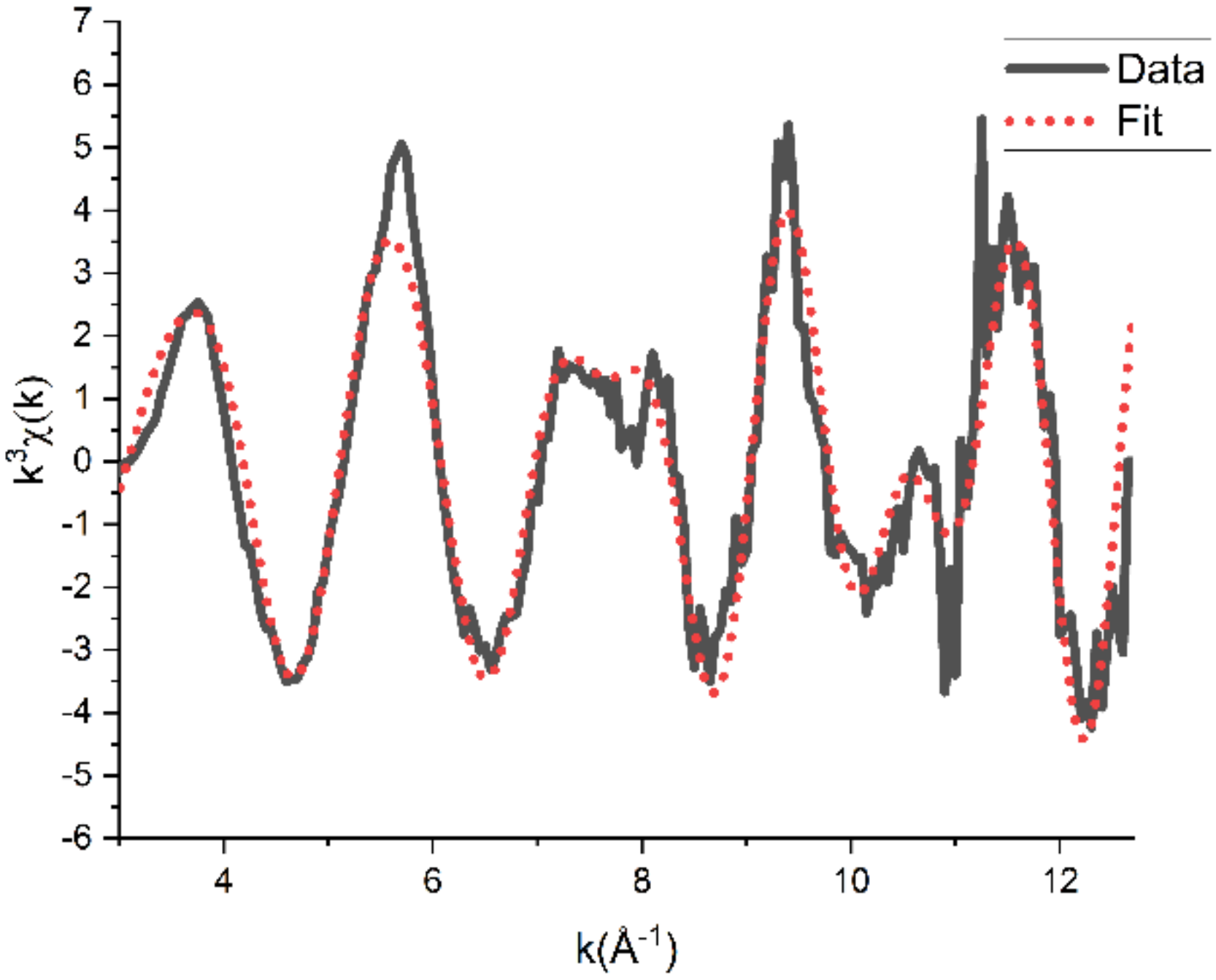

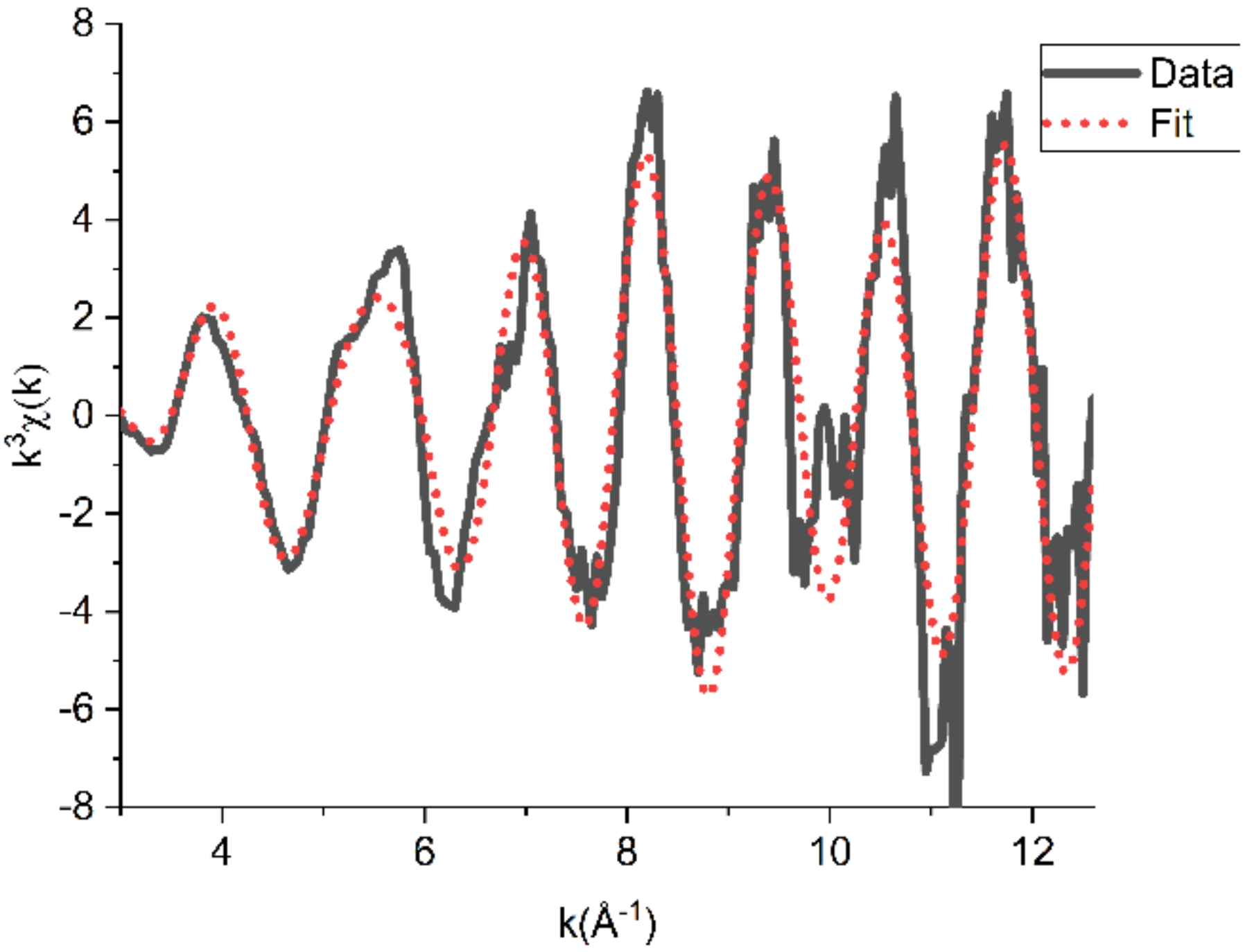

*Figure **S5**. Isolated EXAFS and fit (k^3 weighting) of Pt/Nb_2O_5 (top) and Pt/TiO_2 (bottom). Note results of the analysis are presented in Table S6.*

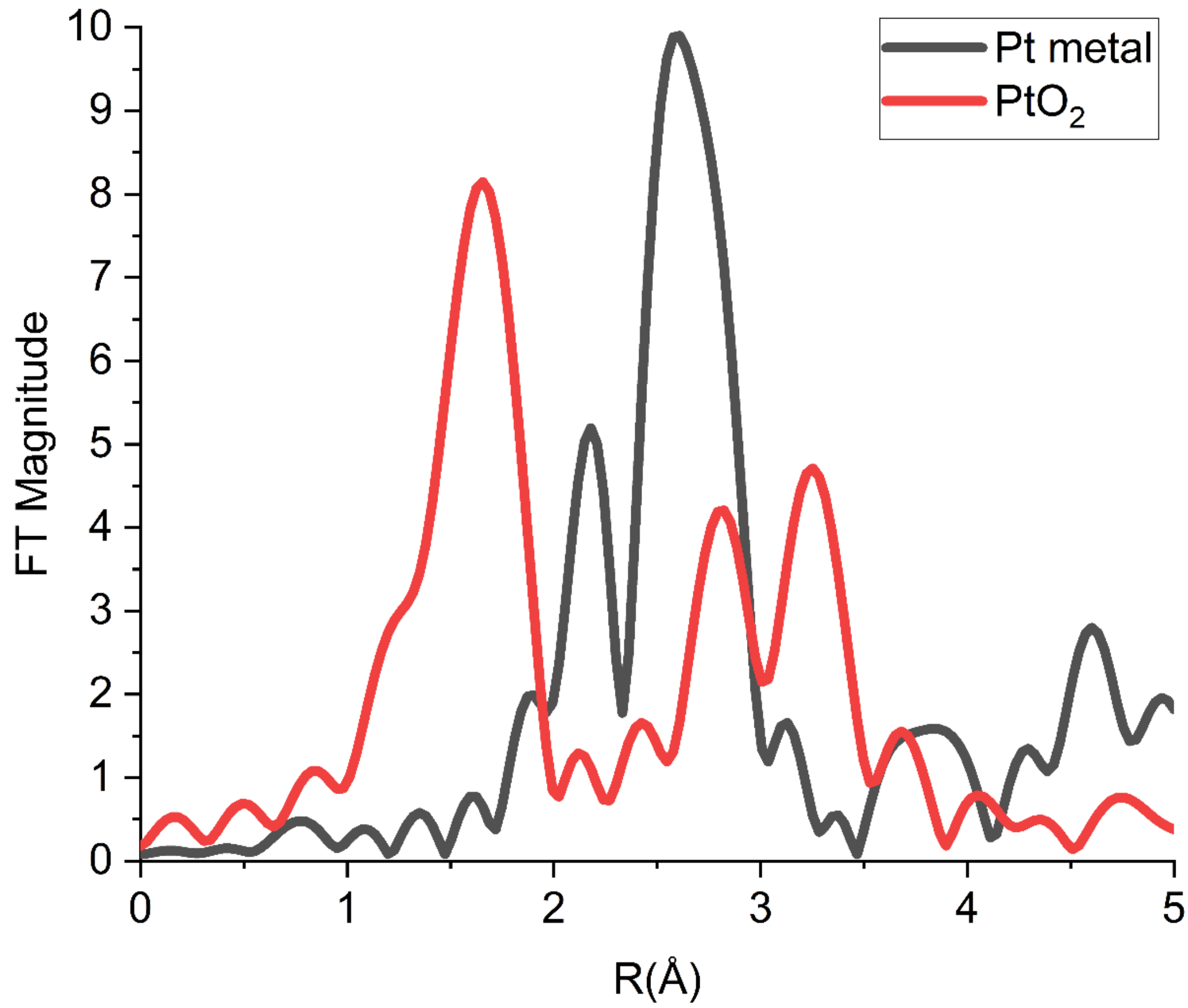

Figure S6: The k^3-weighted Fourier transform spectra for Pt foil and PtO_2 for reference.

Table S5. Results of linear combination fit analysis from the results in figure 4(a)

Sample name	Pt	PtO	PtO_2
0.2 % Pt/Nb_2O_5	50 ± 7	0	50 ± 10
0.2% Pt/TiO_2	79 ± 3	14 ± 6	8 ± 4

Table S6. Results of fit analysis from the results in figure 3.7. (b), (c), (d), and (e)

Parameters	0.2%Pt/TiO_2	0.2%Pt/Nb_2O_5
CN_{Pt-O}	0.7 ± 0.4	2.2 ± 0.4
R_{Pt-O}	1.95 ± 0.03	2 ± 0.01
$\square_{Pt-O}$	0.004 ± 0.008	0.002 ± 0.001
CN_{Pt-Pt}	7 ± 1	1.5 ± 0.9
R_{Pt-Pt}	2.74 ± 0.01	2.77 ± 0.02

σ$_{\text{Pt-Pt}}$	0.006 ± 0.0001	0.004 ± 0.003
ΔE	7 ± 2	13 ± 2
R_{factor}	0.03	0.03

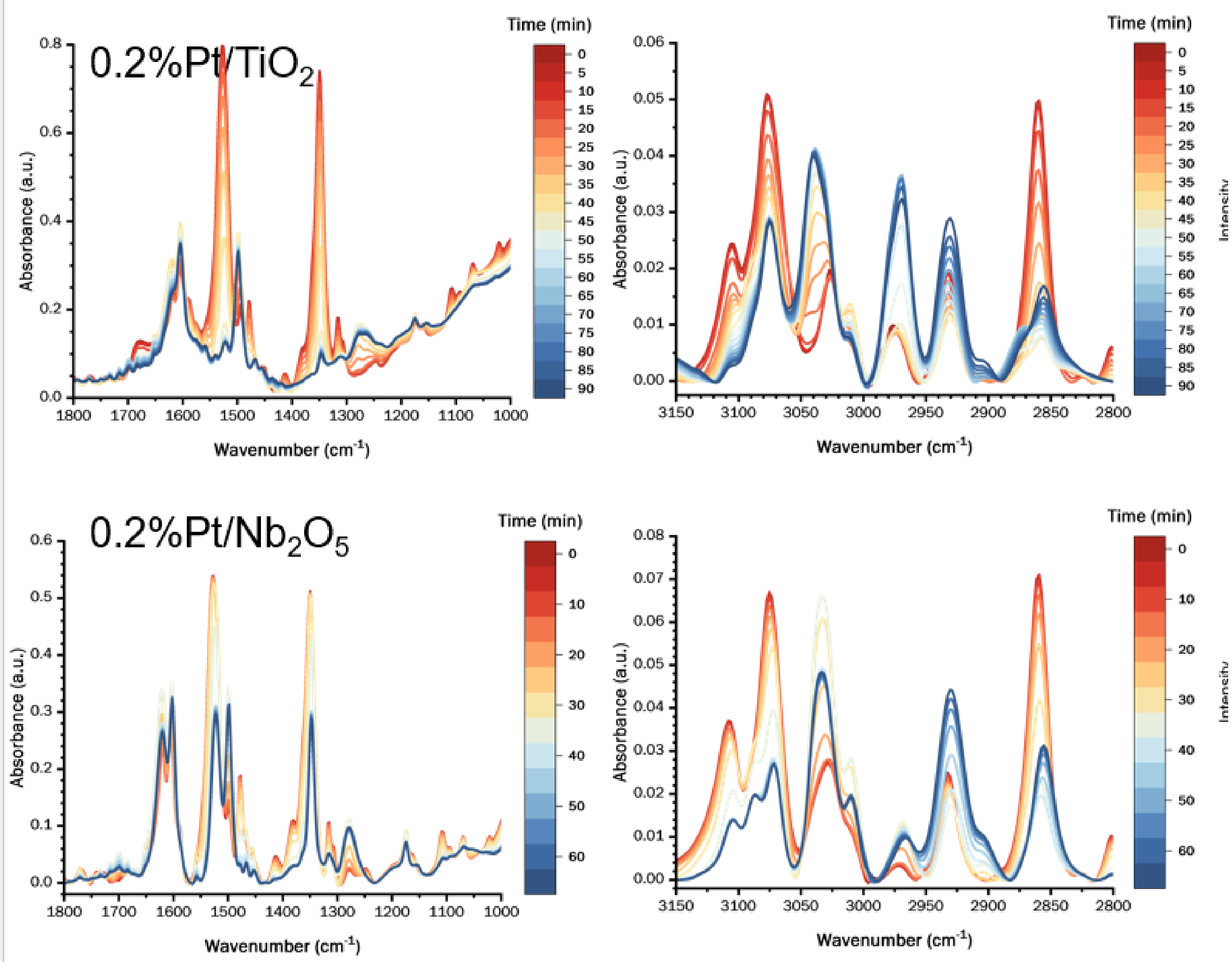

Figure S7. In situ DRIFTS spectra during the hydrogenation of an equimolar mixture of nitrobenzene and styrene over 0.2 wt.% Pt/TiO_2 (top) and 0.2 wt.% Pt/Nb_2O_5 (bottom) at 40 °C

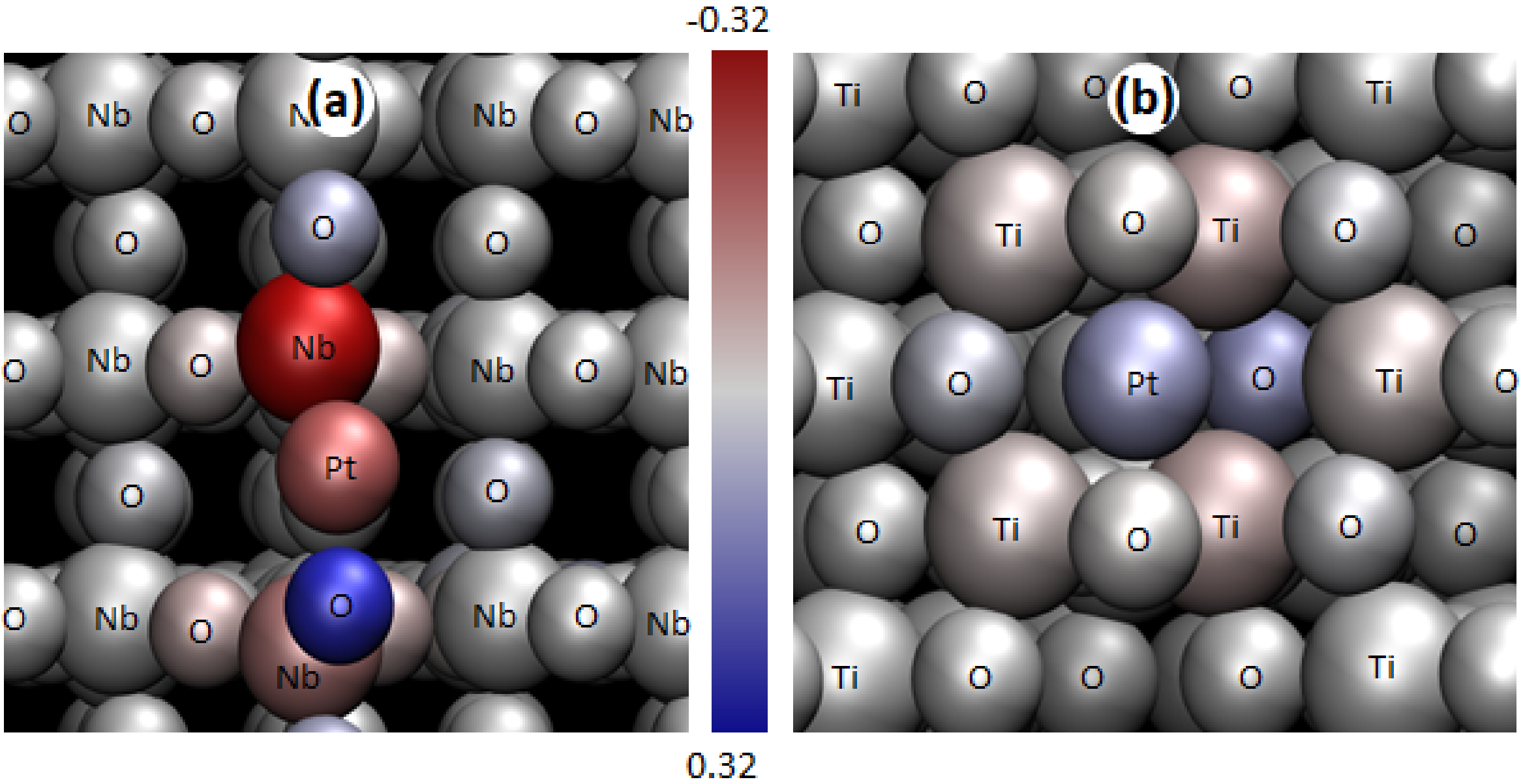

Figure S8: *DFT calculated changes in charge distribution (Δq_{atom}) at support surfaces following the deposition of a Pt atom on (a) Nb_2O_5, and (b) TiO_2. A scale bar is given for the colour scheme, with red and blue representing net charge loss and accumulation, respectively.*

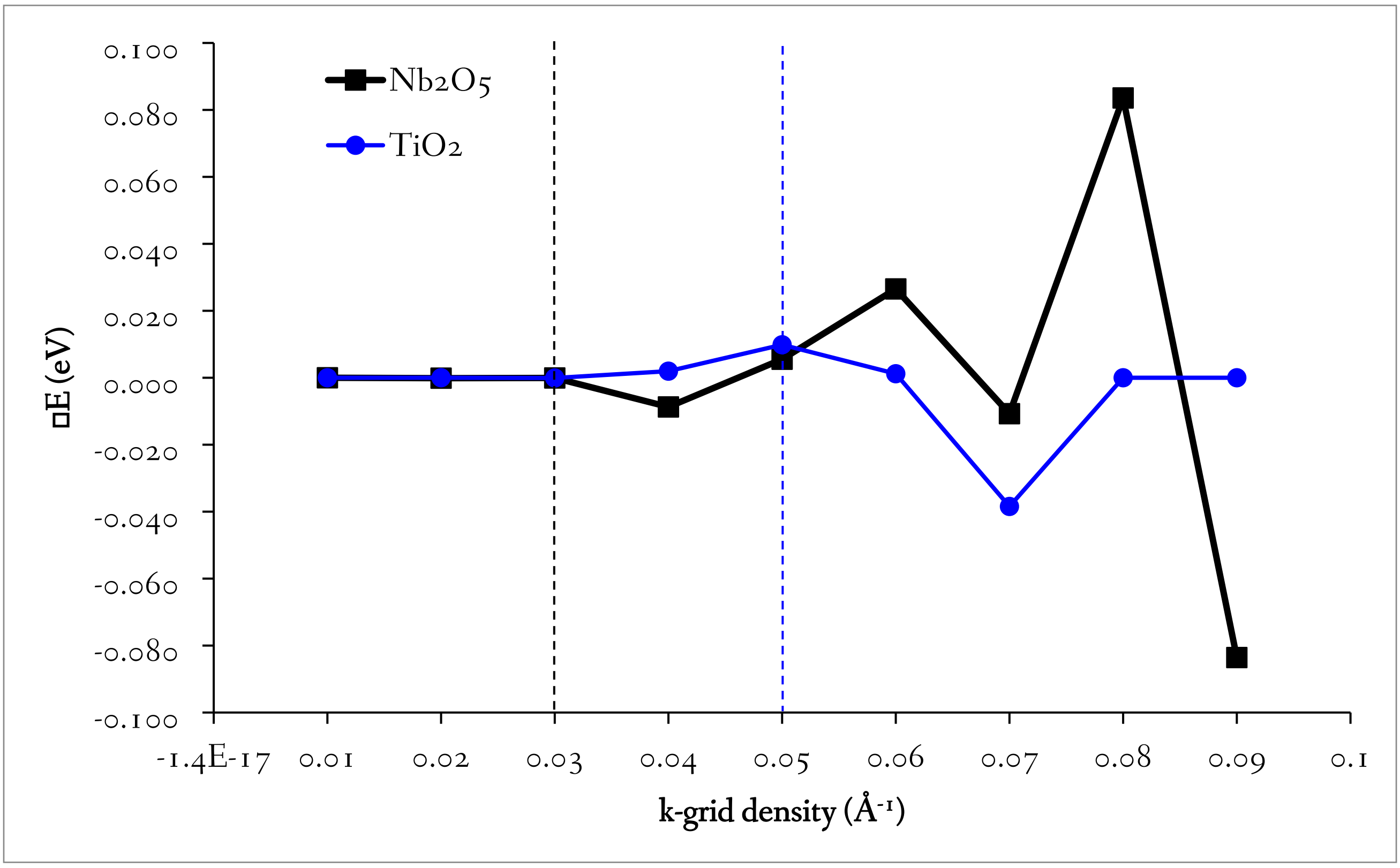

Figure S9: Change in energy (Δ*E*, eV) plotted as a **k**-grid density ($Å^{-1}$) function for TiO_2 and Nb_2O_5 bulk, with the dashed lines showing respective cut-offs.

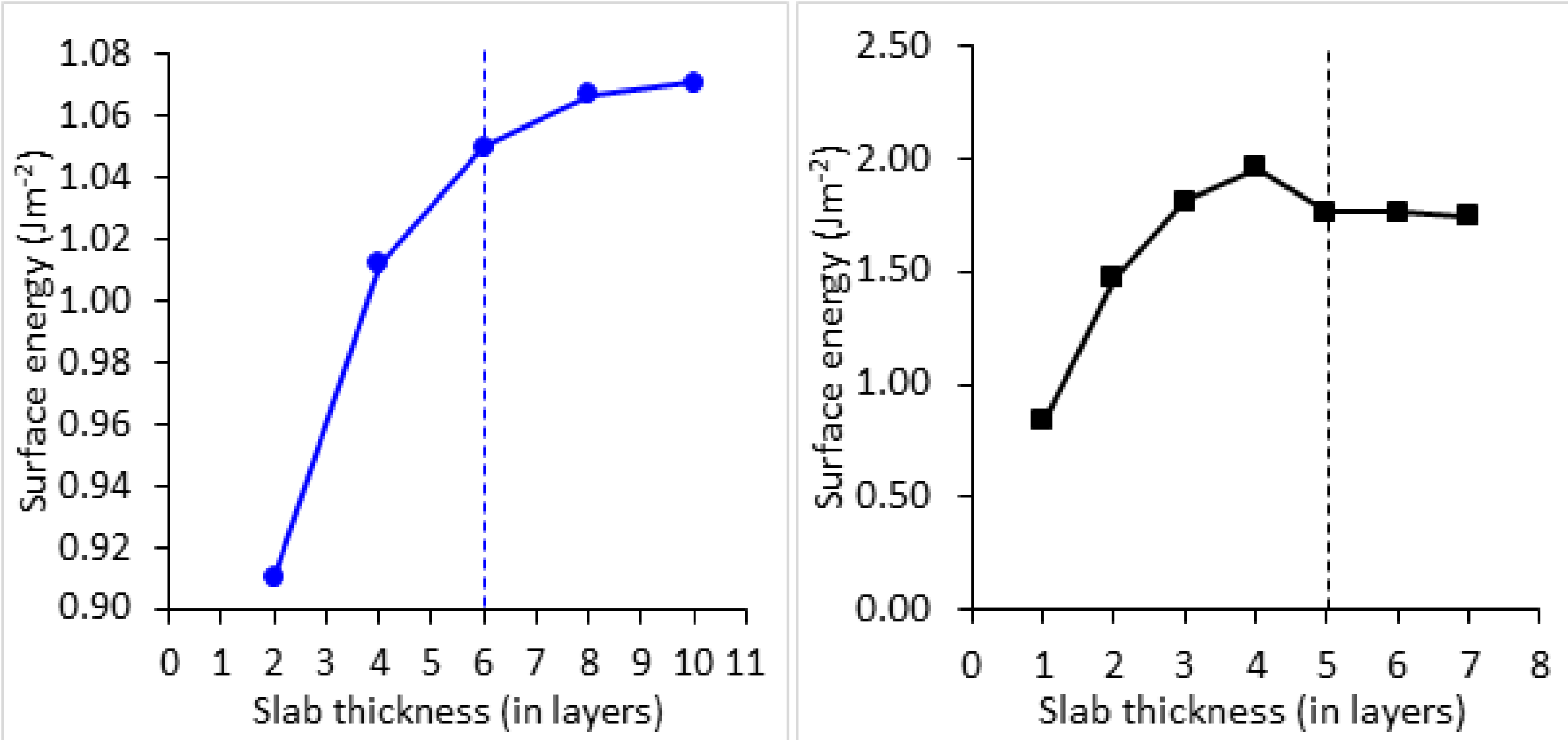

Figure S10: Plot of surface energy (Jm^{-2}) as a function of slab thickness (in layers) with the dashed lines showing cut-off for (001) TiO_2 (top) and (001) Nb_2O_5 (bottom) calculated as ($E_{thick,slab}$ - n∙E_{bulk})/2A, where $E_{thick,slab}$ is the energy calculated for different layer slab in the presence of 20Å vacuum, E_{bulk} is the energy of the unit cell of the metal oxide, n is the number of units of unit cell in the layered slab, and A is the area of the layered slab available for adsorption.

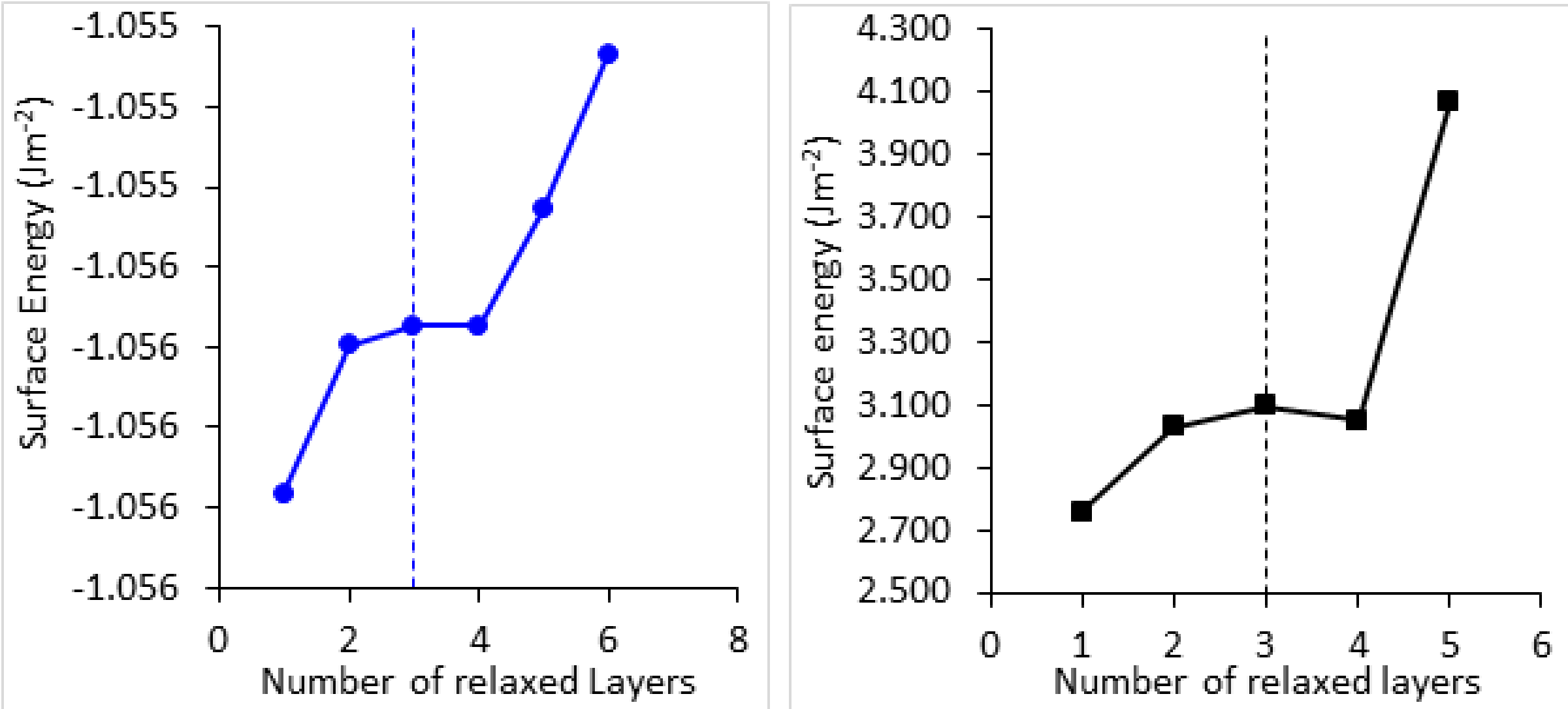

Figure S11: Plot of surface energy (Jm^{-2}) as a function of the number of relaxed layers in the slab with the dashed lines showing cut-off for (001) TiO_2 (top) and (001) Nb_2O_5 (bottom).

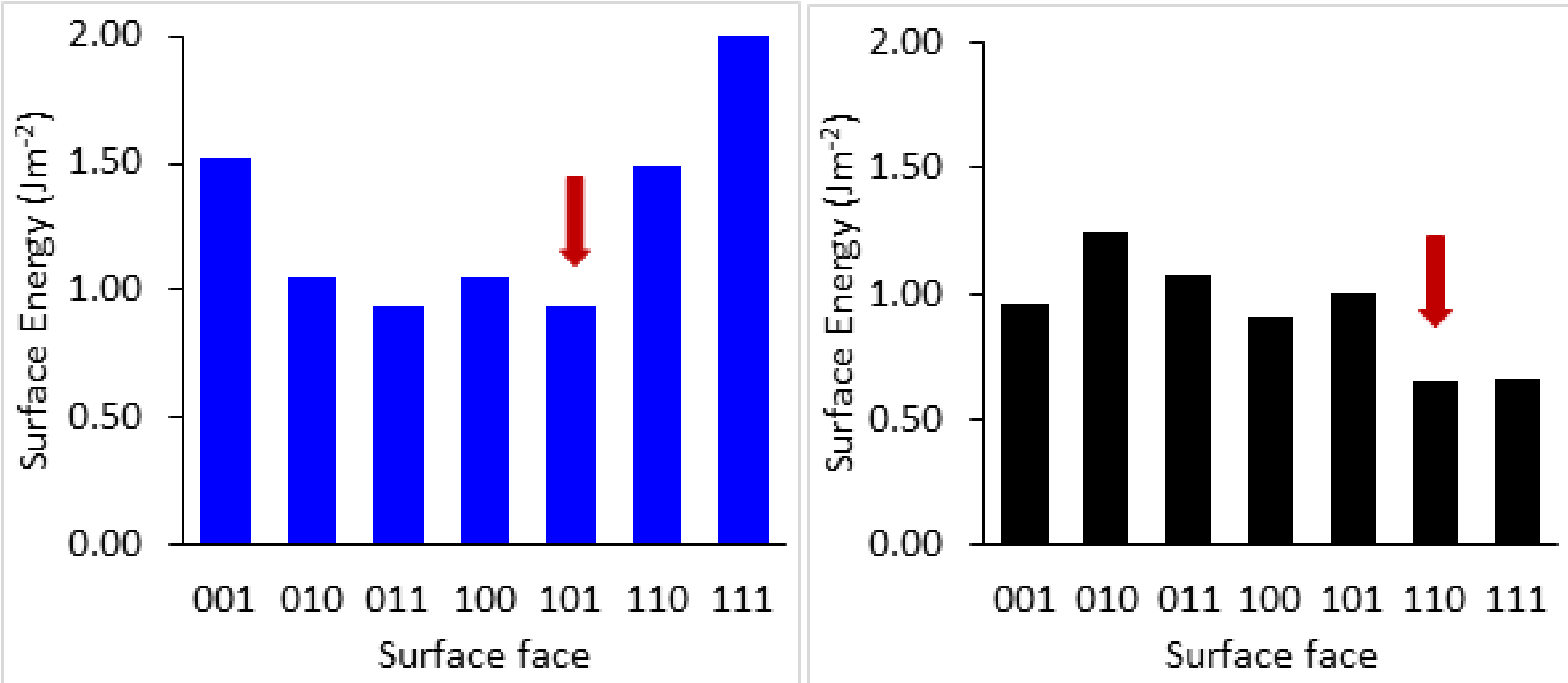

Figure S12: Surface energy plotted as a function of facet for TiO_2 (top) and Nb_2O_5 (bottom) with red arrows highlighting the most stable faces ((101) for TiO_2 and (110) for Nb_2O_5) by red arrows.

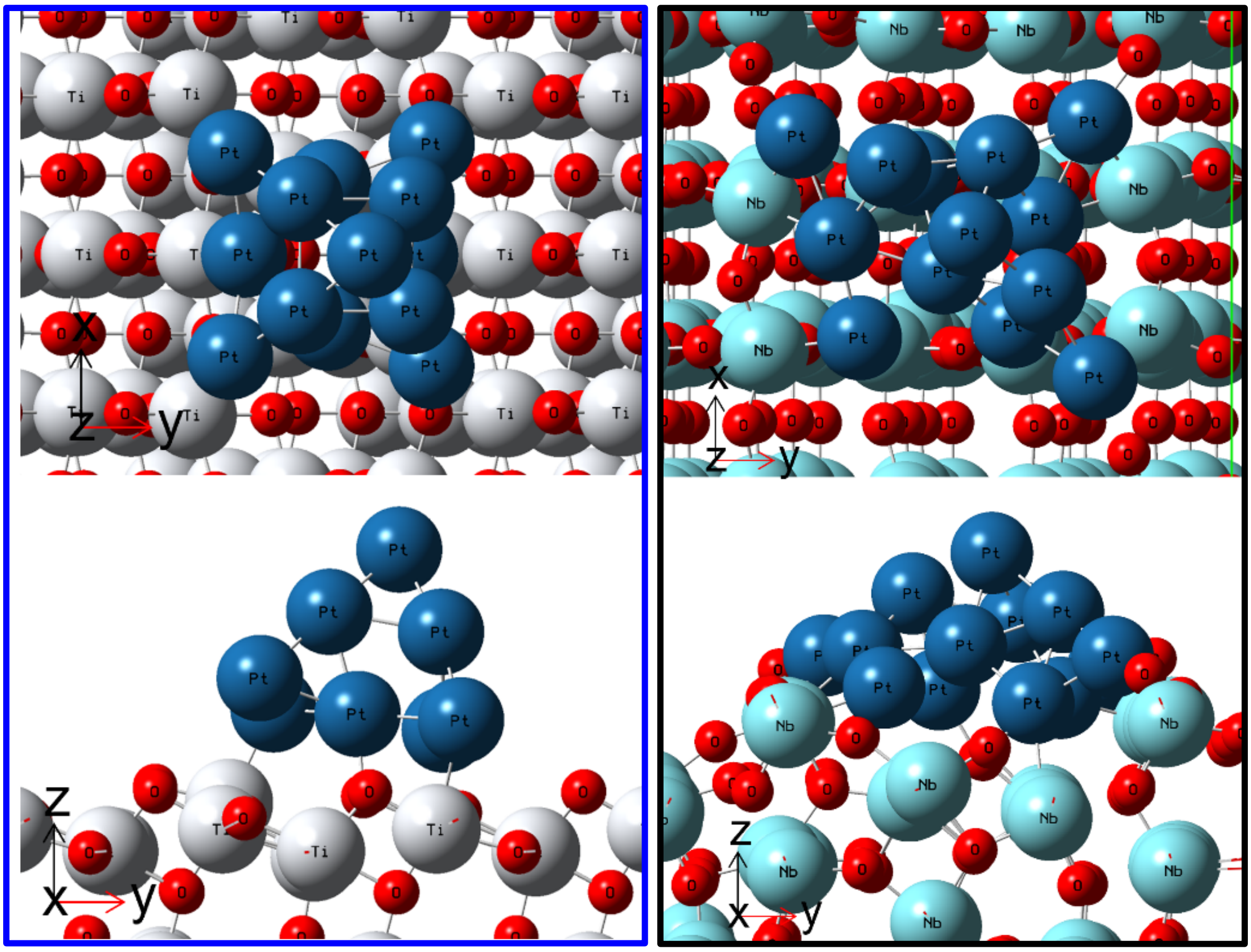

Figure S13: The final geometry of Pt_{13}/TiO_2 (left) and Pt_{13}/Nb_2O_5(right) after relaxation. The structures are uploaded to the NOMAD repository, and can be accessed using the upload id Z5_ECjegSLSUPFd2DYzzrw.

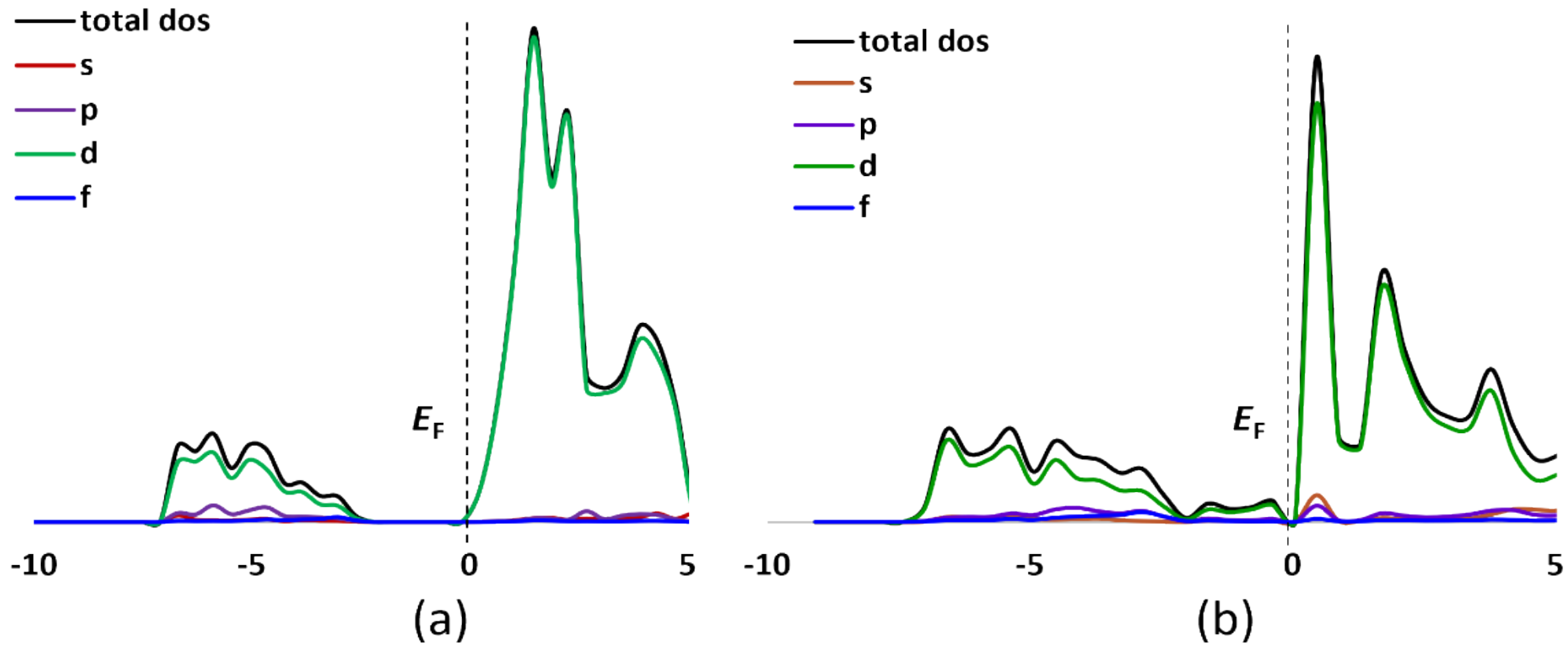

Figure S14: DFT calculated density of states (DOS) and atom-projected DOS (PDOS) calculated for the cations at the support/Pt_{13} interface for (a) Ti metal, and (b) Nb metal.

References:

1 Macino, M. *et al.* Tuning of catalytic sites in Pt/TiO_2 catalysts for the chemoselective hydrogenation of 3-nitrostyrene. *Nature Catalysis* **2**, 873-881(2019).

2 Brett, G.L., *et al.* Selective Oxidation of Glycerol by Highly Active Bimetallic Catalysts at Ambient Temperature under Base-Free Conditions[‡]. Angew. Chem. Int. Ed., 50: 10136-10139 (2011).

3 Peneau, V. *et al.* Selective catalytic oxidation using supported gold–platinum and palladium–platinum nanoalloys prepared by sol-immobilisation. Physical Chemistry Chemical Physics, 15, 10636 – 10644 (2013).

4 https://www.colaver.it/en/reattori-incamiciati-con-collo-svl

5 Wei, J. N., Belanger, D., Adams, R. P. & Sculley, D. Rapid Prediction of Electron–Ionization Mass Spectrometry Using Neural Networks. *ACS Central Science* **5**, 700-708 (2019).

6 Stein, S. E. & Scott, D. R. Optimization and testing of mass spectral library search algorithms for compound identification. *Journal of the American Society for Mass Spectrometry* **5**, 859-866 (1994).

7 Bergeret, G. & Gallezot, P. in *Handbook of Heterogeneous Catalysis* 738-765.

8 Ravel, B. & Newville, M. ATHENA, ARTEMIS, HEPHAESTUS: data analysis for X-ray absorption spectroscopy using IFEFFIT. *Journal of Synchrotron Radiation* **12**, 537-541 (2005).

9 Hjorth Larsen, A. *et al.* The atomic simulation environment—a Python library for working with atoms. *Journal of Physics: Condensed Matter* **29**, 273002 (2017).

10 Blum, V. *et al.* Ab initio molecular simulations with numeric atom-centered orbitals. *Computer Physics Communications* **180**, 2175-2196 (2009).

11 Perdew, J. P., Burke, K. & Ernzerhof, M. Generalized Gradient Approximation Made Simple. *Physical Review Letters* **77**, 3865-3868 (1996).

12 Pinto, M. B., Soares, A. L., Jr., Mella Orellana, A., Duarte, H. A. & De Abreu, H. A. Structural, Electronic, and Thermodynamic Properties of the T and B Phases of Niobia: First-Principle Calculations. *The Journal of Physical Chemistry A* **121**, 2399-2409, (2017).

13 Mancardi, G., Hernandez Tamargo, C., Terranova, U. & de Leeuw, N. H. Calcium Phosphate Deposition on Planar and Stepped (101) Surfaces of Anatase TiO_2: Introducing an Interatomic Potential for the TiO_2/Ca-PO_4/Water Interface. *Langmuir* **34**, 10144-10152 (2018).

14. Hensley, A., Ghale, K., Rieg, C., Dang, T., Anderst, E., Studt, F., Campbell, C. T., McEwen, J., Xu. Y., DFT-Based Method for More Accurate Adsorption Energies: An Adaptive Sum of Energies from RPBE and vdW Density Functionals. J. Phys. Chem. C 2017, 121, 9, 4937–4945.

15 Tkatchenko, A. & Scheffler, M. Accurate Molecular Van Der Waals Interactions from Ground-State Electron Density and Free-Atom Reference Data. *Physical Review Letters* **102**, 073005 (2009).

16 van Lenthe, E., Baerends, E. J. & Snijders, J. G. Relativistic total energy using regular approximations. *The Journal of Chemical Physics* **101**, 9783-9792 (1994).

17 Shannon, D. F., Conditioning of quasi-Newton methods for function minimization Math. Comp. **24**, 647-656 (1970).

18 Broyden, C. G. The Convergence of a Class of Double-rank Minimization Algorithms: 2. The New Algorithm. *IMA Journal of Applied Mathematics* **6**, 222-231(1970).

19 Kusama, H., Orita, H. & Sugihara, H. TiO_2 Band Shift by Nitrogen-Containing Heterocycles in Dye-Sensitized Solar Cells: a Periodic Density Functional Theory Study. *Langmuir* **24**, 4411-4419 (2008).

20 Howard, C. J., Sabine, T. M. & Dickson, F. Structural and thermal parameters for rutile and anatase. *Acta Crystallographica Section B* **47**, 462-468 (1991).

21 Kato, K. & Tamura, S. Die Kristallstruktur von T-Nb_2O_5. *Acta Crystallographica Section B* **31**, 673-677 (1975).

22 Imaoka, T. *et al.* Magic Number Pt13 and Misshapen Pt_{12} Clusters: Which One is the Better Catalyst? *Journal of the American Chemical Society* **135**, 13089-13095 (2013).
